# Transcriptomic and Metabolomic Insights Into the Prebiotic Potential of Camellia Seed Oil for Enhancing *Akkermansia muciniphila* Proliferation In Vitro

**DOI:** 10.1002/fsn3.4637

**Published:** 2024-12-05

**Authors:** Xi Chen, Yong Zhu, Muhammad Mazhar, Likang Qin

**Affiliations:** ^1^ Key Laboratory of Plant Resource Conservation and Germplasm Innovation in Mountainous Region (Ministry of Education), College of Life Sciences/Institute of Agro‐Bioengineering Guizhou University Guiyang China; ^2^ Department of Laboratory Medicine Affiliated Jinyang Hospital of Guizhou Medical University Guiyang China; ^3^ School of Liquor and Food Engineering Guizhou University Guiyang China

**Keywords:** *Akkermansia muciniphila*, camellia (*Camellia oleifera*) seed oil, metabolomics, prebiotics, transcriptomics

## Abstract

Camellia seed oil (CSO), a potential prebiotic agent, can significantly increase the relative abundance of *Akkermansia muciniphila* ( *A. muciniphila* ) in mice gut microbiota following oral administration, this study aims to investigate the enhancing effect in vitro. The results showed that after 24‐h co‐cultivation with 0.5% (v/v) CSO, the growth of *A. muciniphila* increased from 11.61 ± 0.04 Log_10_CFU/mL to 12.17 ± 0.10 Log_10_CFU/mL (*p* < 0.05), accompanied by a reduction in the oxidation–reduction potential (ORP) value of the media from −126.67 ± 1.78 mV to −117.33 ± 0.72 mV (*p* < 0.05). Additionally, squalene and (+)‐α‐tocopherol, bioactive compounds present in CSO, were found to promote *A. muciniphila* proliferation (squalene OD_600_: 1.086 ± 0.002, tocopherol OD_600_: 1.100 ± 0.003, DMSO control OD_600_: 0.991 ± 0.003, *p* < 0.0001). Transcriptomic and metabolomic profiling revealed 464, 121, and 194 differentially expressed genes (DEGs) and 212, 160, and 156 differentially expressed metabolites (DEMs) in *A. muciniphila* co‐cultivated with CSO after 4, 16, and 24 h, respectively (*p* < 0.05). The upregulated DEGs and DEMs were primarily enriched in pathways associated with energy generation (e.g., *gap*, *icd*, *sucC*, *GOZ73_RS04175*, succinate, phosphoenolpyruvate), nucleotide metabolism (e.g., *mazG,* deoxyguanosine), amino acid metabolism (e.g., *argF*, *metK,* L‐tyrosine), translation (e.g., *rplO*, *rpmC*), and environmental adaptation (e.g., *murA*, *katE*, reduced nicotinamide adenine dinucleotide). These findings suggest that various bioactive compounds present in CSO exhibit prebiotic effects on the in vitro proliferation of *A. muciniphila* by facilitating nutrient utilization and environmental adaptation. This study provides insights into the extended utilization of CSO.

## Graphical abstract

Camellia seed oil is rich in bioactive compounds, which could promote the in vitro proliferation of *Akkermansia muciniphila* by reducing the oxidation–reduction potential of the media and activating the metabolism pathways involving energy and nucleic acid metabolism, protein synthesis, and environmental adaptation, thereby facilitating the nutrient utilization and cell replication.

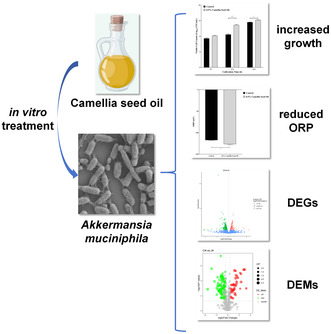

## 1 Introduction

Probiotics, defined as live microorganisms that confer health benefits to the host when consumed in adequate amounts (Hill et al. 2014), have become ubiquitous in the global food industry. The increasing recognition of probiotics' importance is reflected in their widespread consumption, making them one of the most commonly used supplements worldwide (Suez et al. 2019). The general endorsement of probiotics by physicians further underscores their significant role in promoting human health (Draper, Ley, and Parsonnet 2017). Interactions between probiotics, hosts, and surrounding microorganisms are triggered through the cell structures or secreted metabolites of probiotics, leading to alterations in the microenvironment, such as competition for nutrients and anchoring sites (Cunningham et al. 2021).

Numerous studies have demonstrated that specific probiotics, such as *Lactobacillus* and *Bifidobacterium*, can exert health benefits in clinical applications, including modulation of the gut microbiota, maintenance of skin health, and treatment of vaginal dysbiosis (Nguyen et al. 2020; Pendharkar et al. 2015). Moreover, *Akkermansia muciniphila* ( *A. muciniphila* ), a recently discovered intestinal bacterium, has gained significant attention from researchers due to its probiotic potential (Derrien et al. 2004). Previous studies have demonstrated that *A. muciniphila* can degrade mucins in the host gut and stimulate the goblet cells to produce mucin, thereby thickening the mucus layer, which contributes to the enhancement of tight junctions in the gut barrier (Derrien, Belzer, and de Vos 2017). Other studies have indicated that *A. muciniphila* exhibits probiotic properties by effectively reversing metabolic disorders, preventing obesity and related complications (Everard et al. 2013), and enhancing sensitivity to immunotherapy (Routy et al. 2018).

Regarding gut health, a balanced and diverse gut microbiota is essential for various physiological functions in the host. A decline in diversity is commonly associated with a higher prevalence of metabolic disorders (Le Chatelier et al. 2013). The modulation of gut microbiota has emerged as a vital factor in maintaining host health, with dietary intervention as an ideal option (Cotillard et al. 2013). One intriguing aspect of this modulation involves the potential enhancement of the relative abundance of *A. muciniphila* in the gut microbiota through oral administration of cranberry extract or dietary polyphenols (Anhê et al. 2016; Anhê et al. 2015). Researchers have indicated that prebiotics, such as oligosaccharides and dietary fibers, are crucial in promoting the proliferation and metabolism of specific microorganisms (Cunningham et al. 2021; Yang et al. 2022). Since most prebiotics are derived from plants, consuming functional vegetable foods has emerged as a common strategy for acquiring these beneficial compounds.

In recent years, studies have highlighted the potential of fruit, such as pomegranate and kiwifruit, or the bioactive compound ellagitannins found in pomegranates, to elevate *A. muciniphila* levels in the human gut microbiota (Blatchford et al. 2017; Henning et al. 2017; Li et al. 2015). Another study has suggested that the consumption of chia seeds could lead to elevated *A. muciniphila* levels in the gut microbiota (Medina‐Vera et al. 2019). Notably, plant nuts and seeds are rich sources of unsaturated fatty acids and other bioactive compounds that may exhibit prebiotic functions on probiotics. For instance, a study demonstrated that the extracted oil from coix seed significantly enhanced the in vitro proliferation of *Limosilactobacillus reuteri* (Yang et al. 2022). Several studies indicated that polyunsaturated fatty acids can be considered a prebiotic substrate, marking a new frontier in the gut microbiota research (Rinninella and Costantini 2022). For instance, a previous study has demonstrated that camellia seed oil can benefit the growth of *A. muciniphila* in the gut microbiota of mice (Chen et al. 2023), suggesting the potential prebiotic functions of camellia seed oil.

Camellia seed oil, derived from the seeds of *Camellia oleifera Abel*, is usually recognized as the “eastern olive oil” due to its compositional and physicochemical similarities to olive oil (Li et al. 2022). Rich in various bioactive compounds, including unsaturated fatty acids, squalene, vitamin E, phytosterols, tea saponins, and polyphenols (Li et al. 2022), camellia seed oil exhibits notable regulatory effects on blood pressure, cardiovascular health, and antioxidant activities (Chou et al. 2018; Guo et al. 2020; Wu et al. 2022). Previous studies have revealed a correlation between camellia seed oil administration and increased levels of *Dubosiella*, *Lactobacillus*, and *Alistipes* in the gut microbiota of mice (Gao et al. 2022). Another in vivo study similarly concluded that treatment with camellia seed oil could increase the relative abundances of *Actinobacteria*, *Coriobacteriaceae*, *Lactobacillus*, and *Anoxybacillus* (Huang et al. 2021). Even though camellia seed oil has attracted significant attention for its health benefits, there is a notable lack of studies investigating its effects on the proliferation of specific probiotics.

In this study, in vitro cultivation of *A. muciniphila* under camellia seed oil treatment was conducted, building upon insights gained from previous research (Chen et al. 2023). The effects of camellia seed oil and its specific bioactive compounds on the proliferation of *A. muciniphila* were explored. Subsequently, RNA sequencing analysis and ultra‐high‐performance liquid chromatography–mass spectrometry (UHPLC–MS) were conducted to identify the differentially expressed genes (DEGs) and differentially expressed metabolites (DEMs) in *A. muciniphila* at various time points during co‐cultivation with camellia seed oil. This study aims to elucidate the prebiotic effects of camellia seed oil on *A. muciniphila* and to provide valuable insights into its potential applications.

## 2 Materials and Methods

### 2.1 Strains and Culture Conditions

The strain *A. muciniphila* (CICC 24917) was sourced from the China Center of Industrial Culture Collection (Beijing, China) and was initially activated by inoculation on blood agar plates at 37°C for 5 days (Figure S1). The working strains underwent two additional rounds of cultivation under anaerobic conditions at 37°C for 48 h in Brain Heart Infusion (BHI) broth supplemented with 0.5 g/L l‐cysteine, 0.44 g/L N‐acetyl‐d‐glucosamine, and 0.6 g/L l‐threonine (Solarbio, Beijing, China).

### 2.2 Characterization of *A. muciniphila*

The cells of *A. muciniphila* were harvested at different time points during continuous shaking anaerobic cultivation (150 rpm), followed by centrifugation at 7000 rpm, 4°C for 5 min, and re‐suspended with phosphate‐buffered saline (PBS) for the optical density (OD) measurement at 600 nm using a spectrophotometer (INESA L5S, Shanghai, China). Gram staining (Solarbio, Beijing, China) and scanning electron microscopy observation (HITACHI SU8100, Tokyo, Japan) were conducted with the bacterial residue obtained during the stationary growth phase (24 h) (Figure S2).

### 2.3 Co‐Cultivation of *A. muciniphila* With Camellia Seed Oil and Its Specific Compounds

Camellia seed oil, purchased from Guizhou Hengshengyuan Agricultural Development Co., Ltd., was aseptically introduced into BHI broth at a final concentration of 0.5% (v/v) (optimal concentration based on preliminary experiments with additive levels of 0.1%, 0.2%, 0.5%, and 1%) for co‐cultivation with *A. muciniphila* under identical conditions. Viable cell counts of *A. muciniphila* were determined by counting colony‐forming units (CFU) on BHI agar. Specifically, bacterial suspensions were subjected to serial 10‐fold dilutions using sterile, anaerobic, and chilled PBS and inoculated onto BHI agar supplemented with 0.5 g/L l‐cysteine, 0.44 g/L N‐acetyl‐d‐glucosamine, and 0.6 g/L l‐threonine. The plates were incubated anaerobically for 5 days at 37°C. Randomly selected colonies were selected for genomic DNA extraction using the Ezup Column Bacteria Genomic DNA Purification Kit (Sangon Biotech, Shanghai, China), followed by genomic DNA sequencing conducted at Sangon Biotech (Shanghai) Co., Ltd.

β‐Sitosterol was obtained from Aladdin Biochemical Technology Co., Ltd (Shanghai, China). Squalene and (±)‐α‐tocopherol were acquired from Sigma‐Aldrich (St. Louis, USA). These compounds were dissolved in dimethyl sulfoxide (DMSO) to achieve concentrations comparable to the natural levels in the camellia seed oil samples (0.2, 0.036, and 0.116 mg/mL, respectively). Subsequently, these compounds were aseptically introduced into BHI broth for co‐cultivation with *A. muciniphila* under identical conditions. The oxidation–reduction potential (ORP) and optical density at 600 nm (OD_600_) were assessed after 24‐h co‐cultivation.

### 2.4 UHPLC–MS Profiling of Camellia Seed Oil

The UHPLC–MS profiling for camellia seed oil was performed as follows: 80 mg of the oil sample was added to 1000 μL of methanol/acetonitrile/H_2_O (2:2:1, v/v) solution, followed by sonication for 30 min. Subsequently, the mixture was allowed to stand at 20°C for 10 min and then centrifuged at 14,000 *g*, 4°C for 20 min to obtain a supernatant, which was then subjected to vacuum‐freeze dehydration. The dehydrated product was re‐dissolved in 100 μL acetonitrile/water (1:1, v/v) solution and centrifuged at 14,000 *g*, 4°C for 15 min. The final supernatant was utilized for UHPLC–MS analysis employing an Agilent 1290 Infinity LC system (Agilent Technologies, Santa Clara, USA) coupled with an AB Triple TOF 6600 MS system (AB SCIEX, Concord, USA).

A 2.1 mm × 100 mm ACQUITY UPLC BEH Amide 1.7 μm column (Waters, Ireland) was employed for hydrophilic interaction chromatography (HILIC) separation using eluent A (25 mM ammonium acetate and 25 mM ammonium hydroxide in water) and eluent B (acetonitrile) in both positive and negative polarity modes. The solvent gradient was programmed as follows: 95% B, 0.5 min; 95%–65% B, 6.5 min; 65%–40% B, 1 min; 40% B, 1 min; 40%–95% B, 0.1 min; 95% B, 2.9 min. Additional conditions included a column temperature of 25°C, a flow rate of 0.5 mL/min, an injection volume of 2 μL, and an autosampler temperature of 4°C.

The ESI source parameters were set as follows: Ion source gas 1, ion source gas 2, and curtain gas were set to 60, 60, and 30 psi, respectively; ion gas temperature, 600°C; spray voltage, 5500 V (positive), −5500 V (negative). MS‐only data were acquired over the m/z range of 60–1000 Da at 0.2 s/spectra. For MS/MS secondary analysis, a data‐dependent acquisition scanning with a high sensitivity mode was employed (m/z range, 25–1000 Da; accumulation time for product ion scan, 0.05 s/spectra; collision energy, 35 ± 15 eV; declustering potential, ± 60 V; exclude isotopes, within 4 Da; candidate ions per cycle, 10). The ProteoWizard software was used to convert raw MS data into mzXML format. Subsequently, the XCMS software conducted peak alignment, retention time correction, and peak area extraction, facilitating the identification of metabolite structures.

### 2.5 Transcriptomic Profiling of *A. muciniphila* During Co‐Cultivation With Camellia Seed Oil

After 4, 16, and 24 h of co‐cultivation with camellia seed oil in BHI broth, *A. muciniphila* samples (marked O4, O16, and O24, respectively) were centrifuged (7000 rpm, 4°C, 5 min) and re‐suspended with sterile, anaerobic, and chilled phosphate‐buffered saline (PBS) after discarding the supernatant, respectively. This step was repeated three times for samples obtained at each time point. Simultaneously, *A. muciniphila* samples devoid of camellia seed oil co‐cultivation were marked J4, J16, and J24, respectively, and subjected to identical centrifugation and re‐suspension procedures. Total RNA extraction from the *A. muciniphila* residue was achieved using the RNAprep Pure Cell/Bacteria Kit (Tiangen, Beijing, China). RNA quality was assessed using 1% agarose gels (voltage, 180 V; time, 16 min) and the RNA Nano 6000 Assay Kit on the Agilent 2100 bioanalyzer (Agilent Technologies, Santa Clara, USA).

The samples with optimal RNA quality were selected for cDNA synthesis. Ribodepletion was conducted using the Ribo‐Zero Plus rRNA Depletion Kit (Illumina, San Diego, USA), and the resulting enriched mRNA was then fragmented. Library construction employed the NEBNext Ultra II Directional RNA Library Prep Kit for Illumina (New England Biolabs, Ipswich, USA). The library fragments were purified with an AMPure XP system (Beckman Coulter, Beverly, USA), followed by an additional round of polymerase chain reaction (PCR) amplification and AMPure XP purification. The constructed libraries were assessed in a Qubit 2.0 fluorometer (Life Technologies, Frederick, USA) and validated with an Agilent 2100 bioanalyzer (Agilent Technologies, Santa Clara, USA). The qualified libraries were sequenced on an Illumina NovaSeq 6000 platform (Illumina, San Diego, USA).

Sequencing reads containing adapters, N bases, and low‐quality reads were excluded to obtain cleaned paired‐end reads. Subsequently, the reads were aligned to the reference genome using the Bowtie2 v2.3.4.3 software. Read counts for each gene were determined using HTSeq v0.6.1 software, and fragments per kilobase of exon model per million mapped fragments (FPKM) were generated. Differential expression analysis was performed using the DESeq R package v1.20.0, and genes with an adjusted *p*‐value (*p*adj) < 0.05 were assigned as differentially expressed. Gene Ontology (GO) and Kyoto Encyclopedia of Genes and Genomes (KEGG) enrichment analyses were performed using clusterProfiler v3.8.1. Gene set enrichment analysis (GSEA) was conducted using gsea v3.0 software.

### 2.6 Verification of Relative Gene Expression via Reverse Transcription Quantitative Polymerase Chain Reaction (RT‐qPCR)

The procedures for both harvesting bacterial residue and total RNA extraction followed the steps employed in transcriptomic profiling. The concentration and purity of extracted RNA were determined using a Nanodrop microvolume spectrophotometer (Thermo Fisher Scientific, Waltham, USA). Subsequently, cDNA synthesis was achieved using the HiScript II 1st strand cDNA synthesis kit (Vazyme Biotech Co., Ltd., Nanjing, China). RT‐qPCR analysis was conducted with iTaq Universal SYBR Green Supermix (Bio‐Rad, Hercules, USA) on an Applied Biosystems 7500 real‐time PCR system (Thermo Fisher Scientific, Foster City, USA) under the following conditions: Initial denaturation at 95°C for 30 s; 40 cycles of denaturation at 95°C for 15 s, followed by annealing/extension at 60°C for 60 s. The *16S rRNA* gene was selected as the housekeeping gene for normalizing target gene expression levels. Relative gene expression was calculated using the $2^{-∆∆C_{t}}$ method. The primers were obtained from Sangon (Sangon Biotech Co., Ltd., Shanghai, China) and listed in Table S1.

### 2.7 Metabolomic Profiling of *A. muciniphila* During Co‐Cultivation With Camellia Seed Oil

The procedures for harvesting *A. muciniphila* residue followed the steps employed for sample collection in transcriptomic profiling. The residues were re‐suspended in 300 μL of chilled 80% methanol and rapidly frozen in liquid nitrogen for 5 min. Subsequently, the samples were thawed on ice, vigorously vortexed for 30 s, and sonicated for 6 min. The samples were centrifuged (5000 rpm, 4°C, 1 min), and the resulting supernatant was subjected to vacuum‐freeze dehydration. The dehydrated product was re‐dissolved in 10% methanol for UHPLC–MS profiling on a Vanquish UPLC system (Thermo Fisher, Germany) coupled with an Orbitrap Exactive HF/Q mass spectrometer (Thermo Fisher, Germany).

A Hypersil gold column (2.1 mm × 100 mm, 1.9 μm particle size, Thermo Fisher, USA) was utilized for chromatographic separation. The positive polarity mode employed eluent A (0.1% formic acid in water) and eluent B (methanol), while the negative polarity mode used eluent A (5 mM ammonium acetate, pH 9.0) and eluent B (methanol). The solvent gradient was programmed as follows: 2% B, 1.5 min; 2%–85% B, 1.5 min; 85%–100% B, 7 min; 100%–2% B, 0.1 min; 2% B, 1.9 min. Additional conditions included a column temperature of 40°C and a flow rate of 0.2 mL/min. For mass spectrometry, in both positive and negative polarity modes, the following conditions were applied: Spray voltage, 3500 V; sheath gas, 35 psi; auxiliary gas, 10 L/min; capillary temperature, 320°C; S‐lens RF level, 60; auxiliary gas heater temperature, 350°C; m/z scan range, 100–1500. The MS/MS secondary analysis utilized a data‐dependent acquisition scanning approach.

The raw MS data were processed utilizing Compound Discoverer 3.3 software (Thermo Fisher, USA), encompassing the peak alignment, peak picking, and quantification for metabolites. Subsequently, the identified peaks were systematically cross‐referenced with the mzCloud, mzVault, and MassList databases to attain precise qualitative and relative quantitative data. KEGG and LIPIDMaps databases further annotated the identified metabolites to elucidate the metabolic pathways.

### 2.8 Statistical Analysis

All experiments were conducted in biological triplicates (*n* = 3), and results are expressed as mean ± standard error of the mean (SEM). Statistical analyses were conducted using SPSS statistics software (version 19.0, IBM, USA) with an unpaired Student's *t* test, with statistical significance set at *p* < 0.05.

## 3 Results

### 3.1 Camellia Seed Oil and Its Specific Compounds Promote the Growth of *A. muciniphila* In Vitro

**FIGURE 1:**
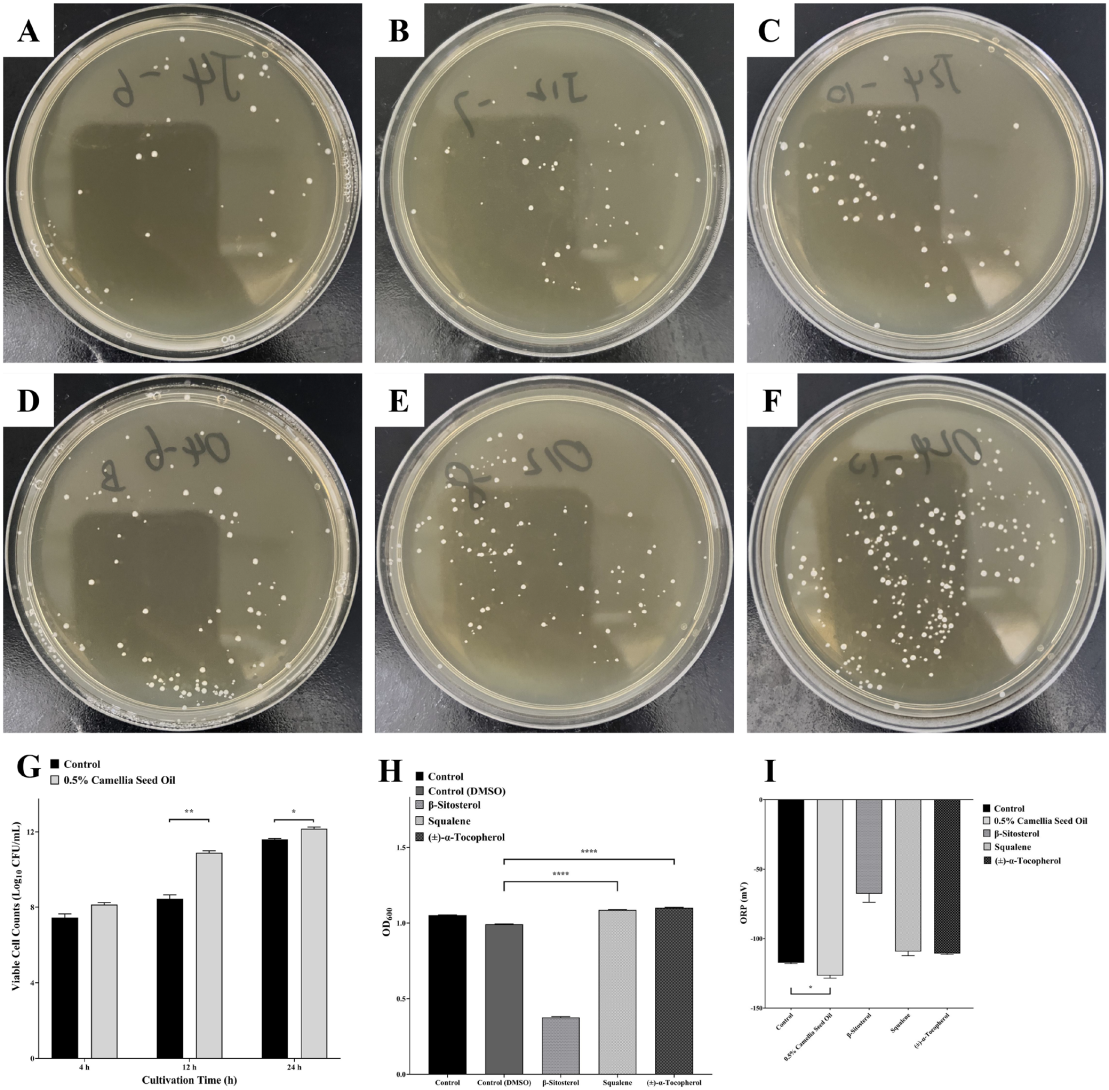
Viable cell counts of *A. muciniphila* in BHI broth after 4, 12, and 24 h, without (A–C) and with (D–F) 0.5% camellia seed oil treatment. The data were visualized in a bar graph (G). (H) OD_600_ values of *A. muciniphila* after 24 h of co‐cultivation with β‐sitosterol, squalene, and (+)‐α‐tocopherol. (I) ORP values after 24 h of co‐cultivation. Data were presented as mean ± SEM (*n* = 3) (*p < 0.05, **p < 0.01, ****p < 0.0001).

After 4, 12, and 24 h of cultivation in BHI broth, *A. muciniphila* suspensions were diluted and inoculated onto BHI agar for an additional 5 day cultivation (Figure 1A–C). The viable cell counts after 4, 12, and 24 h of cultivation were 7.45 ± 0.19 Log_10_CFU/mL, 8.44 ± 0.21 Log_10_CFU/mL, and 11.61 ± 0.04 Log_10_CFU/mL, respectively. In the presence of 0.5% (v/v) camellia seed oil in BHI broth (Figure 1D–F), the viable cell counts of *A. muciniphila* were increased to 8.14 ± 0.11 Log_10_CFU/mL, 10.89 ± 0.11 Log_10_CFU/mL, and 12.17 ± 0.10 Log_10_CFU/mL, respectively. The difference was statistically significant after 12 and 24 h of co‐cultivation with camellia seed oil (*p* < 0.05) (Figure 1G). Genomic DNA sequencing results of randomly selected colonies on BHI agar indicated the genetic purity of *A. muciniphila* co‐cultivated with camellia seed oil (Figure S3 and Tables S2 and S3). Additionally, both squalene and (+)‐α‐tocopherol, which are bioactive compounds present in camellia seed oil, significantly enhanced the biomass of *A. muciniphila* after 24‐h co‐cultivation (squalene OD_600_: 1.086 ± 0.002, tocopherol OD_600_: 1.100 ± 0.003, DMSO control OD_600_: 0.991 ± 0.003, *p* < 0.0001). However, β‐sitosterol did not exhibit such an effect (Figure 1H). The application of 0.5% camellia seed oil in BHI broth led to reduced ORP values of the media after 24‐h co‐cultivation (−126.67 ± 1.78 mV vs. −117.33 ± 0.72 mV, *p* < 0.05). In contrast, none of the three specific compounds present in camellia seed oil exhibited a similar effect (Figure 1I).

The total ion chromatogram generated by UHPLC–MS profiling of camellia seed oil is depicted in Figure S4. Table S4 enumerates compounds present in camellia seed oil with matching scores exceeding 0.7, encompassing various bioactive substances, such as zerumbone, asiatic acid, betaine, gramine, etc.

### 3.2 Transcriptomic Profiling of *A. muciniphila* During Co‐Cultivation With Camellia Seed Oil

The quality of both the RNA extraction and transcriptomic sequencing data strictly adhered to the requirements for subsequent transcriptomic profiling (Tables S5 and S6). Significant DEGs were determined with a threshold of *p*adj ≤ 0.05 and |log2FoldChange| ≥ 0.0 through the DESeq R package v1.20.0. The transcriptomic datasets are accessible in the NCBI BioProject repository under the accession number PRJNA1086711.

**FIGURE 2:**
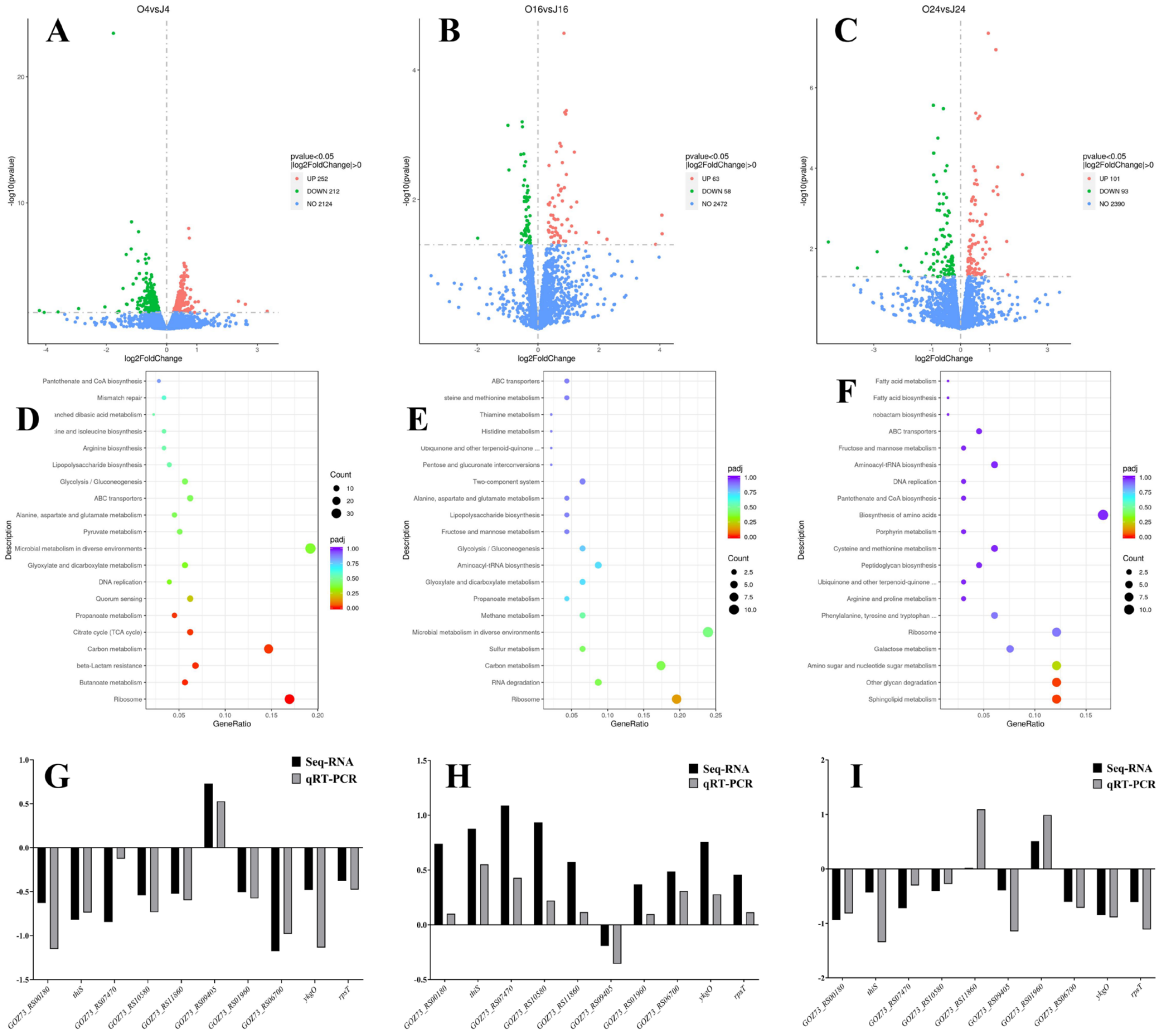
Volcano plots of DEGs (A–C) and KEGG enrichment diagrams (D–F) of *A. muciniphila* during co‐cultivation with camellia seed oil at different time points (*n* = 3): (A, D) O4 vs. J4 (4‐h cultivation), (B, E) O16 vs. J16 (16‐h cultivation), and (C, F) O24 vs. J24 (24‐h cultivation). (G–I) RT‐qPCR verification results of randomly selected genes of *A. muciniphila* during co‐cultivation with camellia seed oil at different time points: (G) O4 vs. J4 (4‐h cultivation), (H) O16 vs. J16 (16‐h cultivation), and (I) O24 vs. J24 (24‐h cultivation).

464 DEGs were identified in *A. muciniphila* after 4‐h co‐cultivation with 0.5% camellia seed oil (O4 vs. J4, corresponding to the lag phase). Among these DEGs, 252 genes were significantly upregulated. In comparison, 212 genes were significantly downregulated (Figure 2A). After 16 h co‐cultivation (O16 vs. J16 group, corresponding to the late exponential phase), 121 DEGs were identified in *A. muciniphila* . Of these DEGs, 63 genes were significantly upregulated, and 58 genes were significantly downregulated (Figure 2B). After 24 h co‐cultivation (O24 vs. J24 group, corresponding to the early stationary phase), a total of 194 DEGs were identified in *A. muciniphila* . Among these DEGs, 101 genes were significantly upregulated, while 93 genes were significantly downregulated (Figure 2C).

Gene Ontology (GO) analysis was performed on DEGs, encompassing three functional categories of biological process (BP), cellular component (CC), and molecular function (MF). The majority of DEGs were enriched in GO terms related to cellular macromolecule biosynthetic process, peptide biosynthetic process, translation, organelle, and DNA binding transcription factor activity (Figure S5). KEGG analysis was further conducted to elucidate the molecular and biological functions of the DEGs. After 4 h co‐cultivation (O4 vs. J4), the DEGs exhibited significant enrichment in the pathways of propanoate metabolism, citrate cycle (TCA cycle), carbon metabolism, butanoate metabolism, and ribosome (Figure 2D). After 16 h co‐cultivation (O16 vs. J16), notable enrichment was observed in the microbial metabolism in diverse environments, carbon metabolism (Figure 2E). After 24 h co‐cultivation (O24 vs. J24), the DEGs were predominantly enriched in the galactose metabolism, amino sugar and nucleotide sugar metabolism, other glycan degradation, and sphingolipid metabolism pathways (Figure 2F).

Based on the KEGG enrichment analysis, GSEA was conducted to address the potential omission of genes exhibiting subtle yet biologically significant expression differences (Subramanian et al. 2005). The gene sets exhibiting significant upregulation in *A. muciniphila* co‐cultivated with camellia seed oil at different time points are presented in Table 1 and Figure S6. These gene sets were primarily associated with basic metabolism, biosynthesis, and environmental adaptation.

**TABLE 1:** GSEA of *A. muciniphila* during co‐cultivation with camellia seed oil.

Cultivation time	Gene set	ID	Normalized enrichment score (NES)
4 h	Arginine biosynthesis	AMU00220	1.344
	Alanine aspartate and glutamate metabolism	AMU00250	1.338
16 h	Ribosome	AMU03010	1.485
	Protein export	AMU03060	1.482
	Quorum sensing	AMU02024	1.307
	Fructose and mannose metabolism	AMU00051	1.295
24 h	Porphyrin metabolism	AMU00860	1.454
	Amino sugar and nucleotide sugar metabolism	AMU00520	1.416
	Biosynthesis of cofactors	AMU01240	1.410
	Antigen nucleotide sugar biosynthesis	AMU00541	1.393
	Other glycan degradation	AMU00511	1.380
	Two‐component system	AMU02020	1.347

The transcriptomic sequencing data were validated by RT‐qPCR. The results were generally consistent with the expression trend of RNA sequencing, indicating the reliability of the transcriptomic sequencing data (Figure 2G–I).

### 3.3 Metabolomic Profiling of *A. muciniphila* During Co‐Cultivation With Camellia Seed Oil

Principal component analysis (PCA) was conducted to explore the metabolites of *A. muciniphila* during co‐cultivation with camellia seed oil in both negative and positive ion modes (Figure S7). The separation observed in the PCA indicated variations in metabolites. Subsequently, partial least‐squares discrimination analysis (PLS‐DA) was performed to further investigate these differences by establishing the relationship model between metabolites and sample classes (Figure S8). All model evaluation parameters, including R2Y and Q2Y, were equal to or close to 1.00, demonstrating the stability and reliability of PLS‐DA and the significant difference of metabolites between groups. Moreover, the permutation test for PLS‐DA was executed to further validate the quality of the model (Figure S9). The results showed that all R2 data were greater than Q2 data, and the intercept between the Q2 regression line and *Y*‐axis was < 0, suggesting the high quality of the PLS‐DA models.

**FIGURE 3:**
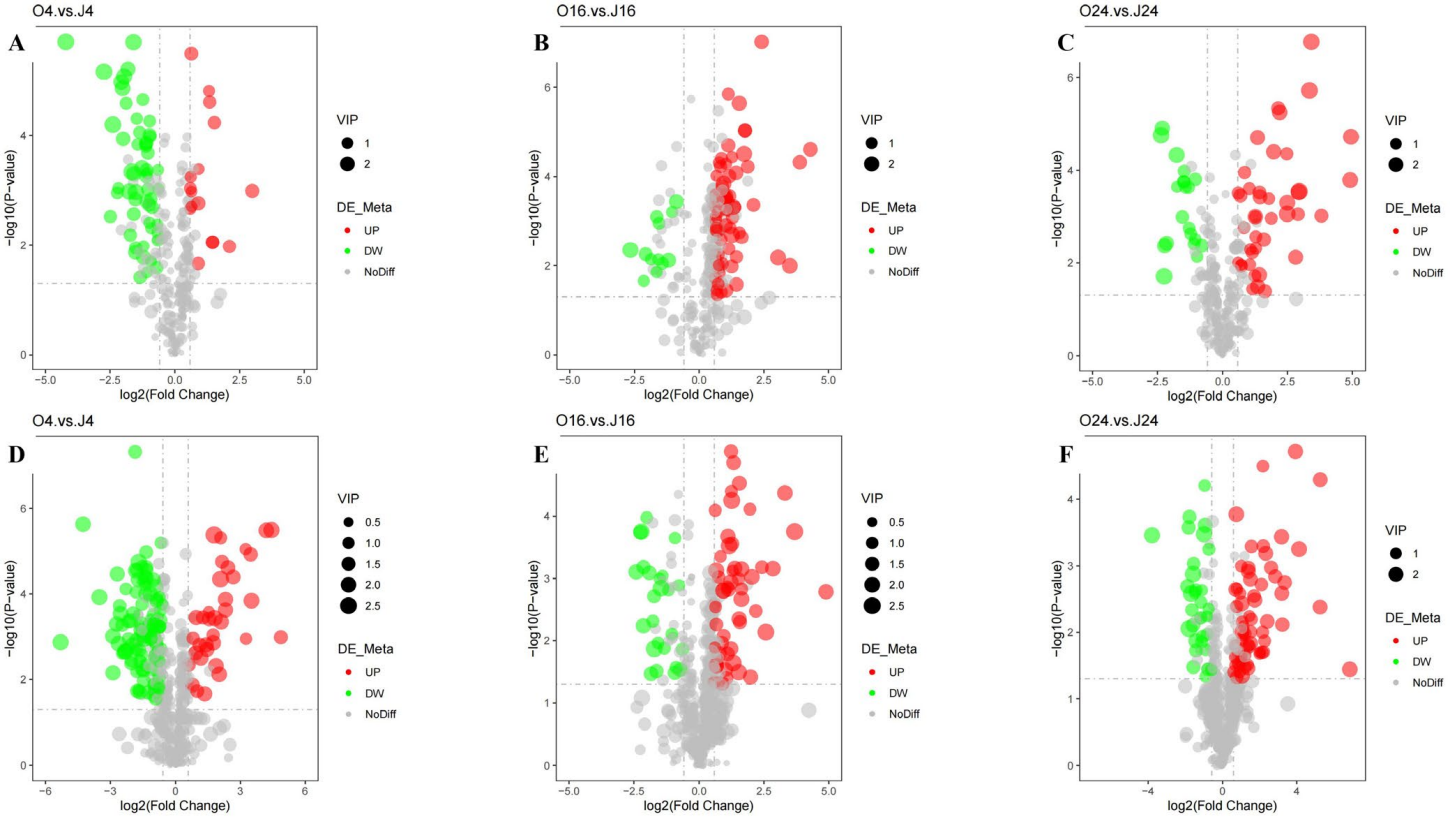
Volcano plots of DEMs of *A. muciniphila* during co‐cultivation with camellia seed oil at different time points (*n* = 3): O4 vs. J4 (4‐h cultivation) in negative (A) and positive (D) ion modes, O16 vs. J16 (16‐h cultivation) in negative (B) and positive (E) ion modes, O24 vs. J24 (24‐h cultivation) in negative (C) and positive (F) ion modes.

**FIGURE 4:**
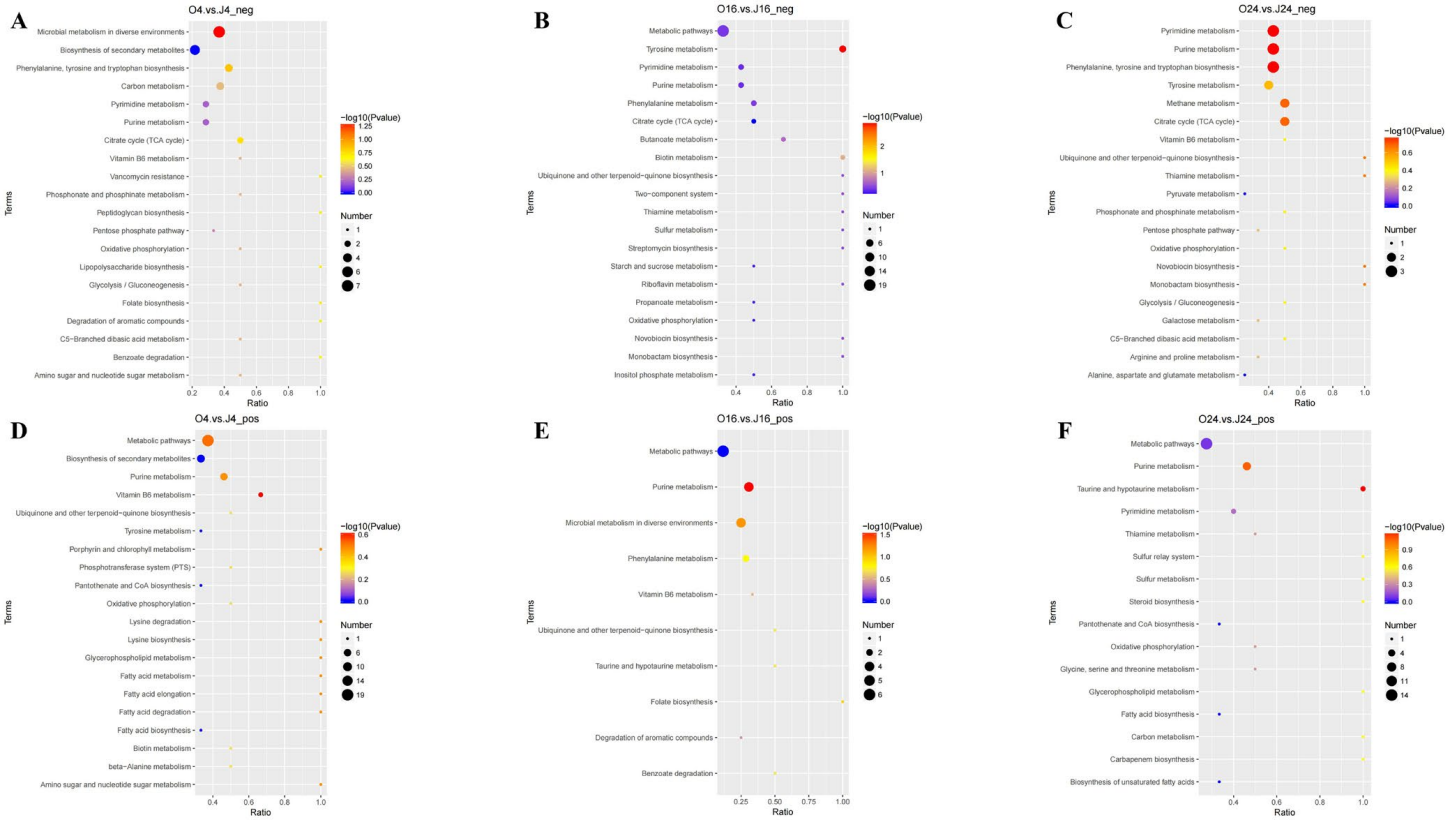
KEGG enrichment diagrams of DEMs of *A. muciniphila* during co‐cultivation with camellia seed oil at different time points (*n* = 3): O4 vs. J4 (4‐h cultivation) in negative (A) and positive (D) ion modes, O16 vs. J16 (16‐h cultivation) in negative (B) and positive (E) ion modes, O24 vs. J24 (24‐h cultivation) in negative (C) and positive (F) ion modes.

DEMs were identified based on variable importance in the projection (VIP) > 1, fold change (FC) > 1.5 or FC < 0.667, and *p* < 0.05. The results, summarized in Table 2, cover various compound classes, including organic acids, amino acids, peptides, benzenoids, and lipids. Figure 3 illustrates the overall distribution of DEMs, with upregulated metabolites represented by red dots and downregulated metabolites by green dots. The top 20 DEMs from each group are displayed in Figure S10. The upregulated metabolites were primarily associated with basic metabolism (e.g., propionyl‐l‐carnitine, succinate, guanosine monophosphate, and flavin adenine dinucleotide), biosynthesis and regulation (e.g., indole‐3‐lactic acid and pantothenic acid), as well as DNA biosynthesis (e.g., ADP‐ribose). KEGG analysis was conducted to investigate the molecular and biological functions of the DEMs (Figure 4). After 4 h of co‐cultivation, the DEMs showed significant enrichment in pathways of the carbon metabolism, biosynthesis of secondary metabolites, TCA cycle, and nucleotide metabolism. After 16 h of co‐cultivation, notable enrichment was observed in the metabolic pathways, TCA cycle, nucleotide metabolism, and butanoate metabolism. After 24 h of co‐cultivation, the DEMs were enriched in the nucleotide metabolism, TCA cycle, and amino acid biosynthesis.

**TABLE 2:** Screening results for DEMs.

Compared samples	Number of total identified metabolites	Number of total significant metabolites	
		Upregulated	Downregulated
O4 vs. J4 neg^a^	285	16	55
O4 vs. J4 pos^b^	525	35	106
O16 vs. J16 neg	285	68	12
O16 vs. J16 pos	525	55	25
O24 vs. J24 neg	285	41	20
O24 vs. J24 pos	525	63	32

^a^ Negative ion mode.

^b^ Positive ion mode.

### 3.4 Integrated Analysis of KEGG Pathway Enrichment in Both Transcriptomics and Metabolomics Data

**FIGURE 5:**
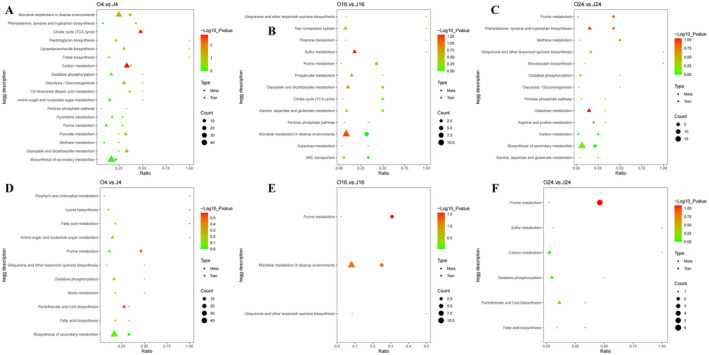
Integrated analysis of transcriptomic and metabolomic data enriched in KEGG pathways. Data were obtained from *A. muciniphila* during co‐cultivation with camellia seed oil at different time points (*n* = 3): O4 vs. J4 (4‐h cultivation) in negative (A) and positive (D) ion modes, O16 vs. J16 (16‐h cultivation) in negative (B) and positive (E) ion modes, O24 vs. J24 (24‐h cultivation) in negative (C) and positive (F) ion modes.

As depicted in Figure 5, DEGs and DEMs were primarily associated with pathways including biosynthesis of secondary metabolites and amino sugar and nucleotide sugar metabolism after 4 h of co‐cultivation with camellia seed oil. Similarly, after 16 and 24 h of co‐cultivation, the pathways were predominantly associated with energy or nucleotide metabolism. The key DEGs and DEMs in the proliferation of *A. muciniphila* co‐cultivated with camellia seed oil are listed in Table 3.

**TABLE 3:** Key DEGs and DEMs in the proliferation of *A. muciniphila* co‐cultivated with camellia seed oil.

Gene (metabolite) name	Log_2_ fold change (time)	Gene description
*acnA*	0.4 (4 h)	Aconitate hydratase AcnA
*icd*	0.28 (4 h)	NADP‐dependent isocitrate dehydrogenase
*sucC*	0.38 (4 h)	ADP‐forming succinate–CoA ligase subunit beta
*GOZ73_RS03580*	0.49 (4 h)	Succinate dehydrogenase cytochrome b subunit
*GOZ73_RS03590*	0.41 (4 h)	Succinate dehydrogenase/fumarate reductase iron–sulfur subunit
*GOZ73_RS11705*	0.26 (4 h)	Fumarate hydratase
*GOZ73_RS11510*	0.63 (4 h)	Malate dehydrogenase
*GOZ73_RS06440*	0.63 (4 h)	Phosphoenolpyruvate carboxykinase
*ppdK*	0.43 (4 h)	Pyruvate phosphate dikinase
*eno*	0.32 (4 h)	Phosphopyruvate hydratase
*gap*	0.38 (4 h)	Type I glyceraldehyde‐3‐phosphate dehydrogenase
*fbaA*	0.36 (4 h)	Class II fructose‐bisphosphate aldolase
*GOZ73_RS07160*	0.34 (4 h)	Galactose mutarotase
*GOZ73_RS10580*	0.93 (16 h)	Ferredoxin
*katE*	0.39 (4 h)	Catalase HPII
*GOZ73_RS10915*	0.58 (4 h)	Methylmalonyl‐CoA mutase family protein
*GOZ73_RS06910*	0.33 (4 h)	Glutamine synthetase III
*gcvP*	0.31 (4 h)	Aminomethyl‐transferring glycine dehydrogenase
*gcvH*	0.48 (4 h)	Glycine cleavage system protein GcvH
*GOZ73_RS02280*	0.57 (24 h)	Family 20 glycosylhydrolase
*GOZ73_RS10590*	0.34 (24 h)	
*GOZ73_RS04175*	0.67 (4 h)	Beta‐N‐acetylhexosaminidase
	0.37 (24 h)	
*GOZ73_RS02135*	0.37 (4 h)	
*GOZ73_RS05495*	0.41 (24 h)	
*GOZ73_RS09305*	0.41 (24 h)	
*murA*	0.31 (24 h)	UDP‐N‐acetylglucosamine 1‐carboxyvinyltransferase
Succinate	0.98 (16 h)	/
Citrate	1.13 (16 h)	/
Phosphoenolpyruvate	1.42 (24 h)	/
D‐Erythrose 4‐phosphate	1.03 (24 h)	/
Reduced nicotinamide adenine dinucleotide (NADH)	1.21 (24 h)	/

## 4 Discussion

Probiotics are crucial in maintaining host health due to the regulatory effects on immune system, metabolic homeostasis, and gut microbiota (Shi et al. 2016). While traditional probiotics, such as *Lactobacillus* and *Bifidobacterium*, have been extensively studied (Nguyen et al. 2020; Pendharkar et al. 2015), the rapid development of bioinformatics tools and next‐generation sequencing techniques has led to the identification of emerging “next‐generation probiotics (NGPs),” which mainly include *Faecalibacterium prausnitzii* , *Bacteroides fragilis* , and *A. muciniphila* (Kaźmierczak‐Siedlecka et al. 2022). The NGPs are extremely sensitive to oxygen and possess health‐promoting potential (Torp et al. 2022). For instance, *Faecalibacterium prausnitzii* is demonstrated to alleviate colitis and enhance the tumor‐suppressive effects of specific immune checkpoint blockade (Gao et al. 2023), and *Bacteroides fragilis* could promote colonic mucosa regeneration in colitis by activating STAT3 signaling pathway (Zhang et al. 2023). Similarly, *A. muciniphila* has garnered particular attention from researchers due to its ability to alleviate metabolic diseases (Everard et al. 2013; Zhai et al. 2019).

The proliferation of probiotics induced by dietary prebiotics has been widely recognized in the field (Cunningham et al. 2021). Furthermore, the diet, as the primary energy source, could induce shifts in gut microbiota within just 24 h (David et al. 2014; Singh et al. 2017). In a previous study (Chen et al. 2023), oral supplementation of camellia seed oil significantly increased the relative abundance of *A. muciniphila* in the gut microbiota of mice, consistent with other studies highlighting the role of unsaturated fatty acids in promoting the relative abundance of *A. muciniphila* (Chaplin et al. 2015; Cunningham et al. 2021; Tian et al. 2016). Consequently, in vitro cultivation of *A. muciniphila* under 0.5% camellia seed oil treatment was conducted in this study to explore the underlying mechanisms of such promotion. Figure 1G illustrates a significant elevation (*p* < 0.05) in the colonies of *A. muciniphila* from 11.61 ± 0.04 Log_10_CFU/mL to 12.17 ± 0.09 Log_10_CFU/mL after 24 h of 0.5% camellia seed oil treatment, suggesting the in vitro growth‐promoting effect of camellia seed oil on *A. muciniphila* .

Camellia seed oil is rich in bioactive compounds (e.g., unsaturated fatty acids, squalene, phytosterols, vitamin E, tea sasanqua saponin, and polyphenols) (Li et al. 2022), making it popular in the food industry and therapeutic applications (Huang et al. 2005; Yang et al. 2015). Hence, three specific bioactive compounds present in camellia seed oil, including β‐sitosterol, squalene, and (+)‐α‐tocopherol, were utilized to treat *A. muciniphila* , aiming to elucidate their promotive effects on the growth of *A. muciniphila* . The results illustrated that both squalene and tocopherol significantly enhanced the proliferation of *A. muciniphila* (Figure 1H) (*p* < 0.0001). Squalene and tocopherol possess various biological functions and exhibit antioxidant activity (Kim et al. 2021; Li et al. 2022; Xiao et al. 2016). However, treatment with either squalene or tocopherol alone did not reduce the ORP values of the media for *A. muciniphila* after 24 h of co‐cultivation (Figure 1I), possibly due to insufficient concentrations of these compounds to produce a noticeable effect on the overall ORP of the media. Nevertheless, camellia seed oil treatment significantly reduced the ORP values of the media for *A. muciniphila* after 24 h of co‐cultivation (*p* < 0.05) (Figure 1I). Subsequently, UHPLC–MS profiling was conducted to investigate the bioactive compounds present in camellia seed oil. The abundant antioxidant compounds, including asiatic acid, alpha‐linolenic acid, brassicasterol, muscone, saikosaponin d, and schizandrin, might contribute to the reduced ORP values of the media (Angel Rincon‐Cervera et al. 2016; Du et al. 2022; Hu et al. 2012; Phung et al. 2020; Ramachandran and Saravanan 2013; Wang, Hicks, and Moreau 2002) (Table S4). Moreover, compounds such as betaine, gramine, madecassic acid, and zerumbone could impact the gut microbiota (Jingjing Du et al. 2021; Qiao et al. 2020; Schuetz et al. 2021), with zerumbone specifically suggested to influence the levels of *A. muciniphila* *in vivo* (Cho, Rhee, and Eom 2020). Therefore, it is speculated that the combined impact of various bioactive compounds present in camellia seed oil induced alterations in the microenvironment within the media, exhibiting a prebiotic‐like function in promoting the proliferation of *A. muciniphila* . Regarding these findings, transcriptomic and metabolomic profiling were conducted. The multi‐omics results indicated that treatment with camellia seed oil could influence various metabolic pathways of *A. muciniphila* , generating diverse DEGs and DEMs.

Energy generation in biological activities supports all subsequent processes. As crucial energy sources, carbohydrates and lipids play vital roles in the metabolism of all organisms, while some bacteria can even utilize fatty acid as the sole carbon source for growth (Fujita, Matsuoka, and Hirooka 2007). In this study, DEGs and DEMS were predominantly enriched in KEGG pathways associated with the TCA cycle, pyruvate metabolism, and glycolysis/gluconeogenesis. However, regarding pathways related to lipid metabolism, camellia seed oil treatment did not significantly promote the proliferation of *A. muciniphila* , except for the sphingolipid metabolism pathway.

**FIGURE 6:**
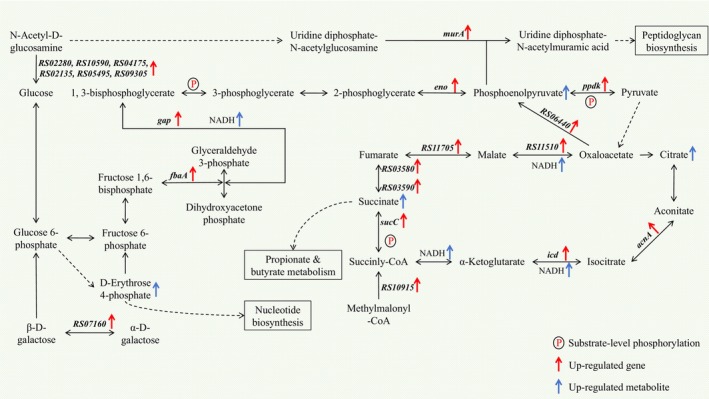
Mechanism of enhanced proliferation of *A. muciniphila* during co‐cultivation with camellia seed oil.

*A. muciniphila* was observed in the lag phase after 4 h of co‐cultivation (Figure S2D), indicating the necessity of synthesizing essential cellular components to adapt to the environment and prepare for exponential growth (Rolfe et al. 2012), a process entailing considerable energy consumption. Compared with the control group (J4), camellia seed oil treatment could enhance the expression of various key genes and metabolites in the proliferation of *A. muciniphila* (Table 3), which is attributed to the abundant monosaccharides and polysaccharides present in camellia seed oil (Luan et al. 2020). Figure 6 shows the mechanism of enhanced proliferation of *A. muciniphila* during co‐cultivation with camellia seed oil. The key genes associated with glycolysis/gluconeogenesis, pyruvate metabolism, and TCA cycle, including *fbaA* (encoding class II fructose‐bisphosphate aldolase), *gap* (encoding type I glyceraldehyde‐3‐phosphate dehydrogenase), *eno* (encoding phosphopyruvate hydratase), *ppdK* (encoding pyruvate phosphate dikinase), *GOZ73_RS06440* (encoding phosphoenolpyruvate carboxykinase), *acnA* (encoding aconitate hydratase AcnA), *icd* (encoding NADP‐dependent isocitrate dehydrogenase), *sucC* (encoding ADP‐forming succinate–CoA ligase subunit beta), *GOZ73_RS03580* (encoding succinate dehydrogenase cytochrome b subunit), *GOZ73_RS03590* (encoding succinate dehydrogenase/fumarate reductase iron–sulfur subunit), *GOZ73_RS11705* (encoding fumarate hydratase), and *GOZ73_RS11510* (encoding malate dehydrogenase), were upregulated in *A. muciniphila* during co‐cultivation with camellia seed oil, resulting in the accumulation of metabolites such as succinate, citrate, phosphoenolpyruvate, and reduced nicotinamide adenine dinucleotide (NADH), as well as an increase in energy reserves through the substrate‐level phosphorylation. In the anaerobic proliferation of *A. muciniphila* , the glycolysis process served as the primary pathway for energy generation, involving two steps of substrate‐level phosphorylation. Likewise, the expression product of *sucC* gene, succinate‐CoA ligase, can hydrolyze succinyl‐CoA to generate either ATP or GTP, representing a unique step of substrate‐level phosphorylation within the TCA cycle. Additionally, succinate‐CoA ligase can also function in the reverse direction for succinyl‐CoA biosynthesis, which is vital during the anaerobic cultivation of *A. muciniphila* due to the suppression of the oxidative route from 2‐oxoglutarate (Nolte et al. 2014).

Moreover, in the activated sphingolipid metabolism pathway, which is crucial for all bacteria owing to their cell membrane composition and cell signaling activities (Simonis and Schubert‐Unkmeir 2018; Zheng et al. 2006), genes encoding glycoside hydrolase family 20 (e.g., *GOZ73_RS04175, GOZ73_RS02135, GOZ73_RS02280, GOZ73_RS10590, GOZ73_RS05495*, and *GOZ73_RS09305*), especially β‐N‐acetylhexosaminidase, were upregulated in *A. muciniphila* co‐cultivated with camellia seed oil. β‐N‐acetylhexosaminidase plays a functional role in the degradation of exogenous glycans, such as N‐acetyl‐D‐glucosamine, thereby benefiting processes such as cell wall modification and nutritional metabolite recycling in bacteria (Liu, Duan, and Yang 2018; Wang et al. 2018). Previous studies have demonstrated that *A. muciniphila* can grow on mucin and mucin‐derived aminosaccharides (e.g., N‐acetylgalactosamine and N‐acetylglucosamine) and produce acetate and propionate (Ottman et al. 2017). In this study, the compound N‐acetyl‐d‐glucosamine was present in BHI media but not in the camellia seed oil. The degradation of N‐acetyl‐d‐glucosamine generated more substrates for glycolysis/gluconeogenesis, TCA cycle, and peptidoglycan biosynthesis. Considering the substantial amount of N‐acetyl‐d‐glucosamine added to the BHI media (0.44 g/L), the enhanced degradation might be one of the main reasons for the increased proliferation of *A. muciniphila* co‐cultivated with camellia seed oil. The absence of significant activation in other lipid metabolism pathways suggested that the enhanced proliferation of *A. muciniphila* may not be solely due to the presence of fatty acids in camellia seed oil but rather to other bioactive compounds. Likewise, the activated metabolism pathways including propanoate metabolism, galactose metabolism, and butanoate metabolism suggested that diverse saccharides and other bioactive compounds present in camellia seed oil could be utilized by *A. muciniphila* to generate energy compared with the control group, leading to enhanced proliferation of *A. muciniphila* during the exponential growth phase (Figure 1G). On the other hand, the upregulated gene *GOZ73_RS10580* (encoding ferredoxin) could enhance the electron transfer activity, thereby further facilitating both anabolic and catabolic metabolism of *A. muciniphila* , including energy generation (Child et al. 2018).

Similarly, significant enrichments were observed in pathways related to critical amino acid metabolism, such as alanine, glycine, glutamate, arginine, and cysteine (Table S7). For instance, the upregulated genes, such as *argF* (encoding ornithine carbamoyltransferase) and *argH* (encoding argininosuccinate lyase), suggested the activation of the arginine biosynthesis pathway, as supported by the GSEA results (Table 1), which was vital for cell survival of bacteria (Ni et al. 2021). Furthermore, the upregulated gene *metK* (encoding methionine adenosyltransferase) can generate S‐adenosyl methionine, an essential cofactor in diverse methylation reactions (Leyn et al. 2014). DNA and RNA methylation could significantly modulate the post‐transcriptional modifications in almost all types of cells, affecting numerous metabolic processes (Motorin and Helm 2022; Nye, Fernandez, and Simmons 2020). The elevated 5‐methylcytosine level in *A. muciniphila* co‐cultivated with camellia seed oil during the lag phase further supported the upregulated methylation reactions. In addition, the generation of cofactor ferredoxin could also benefit from the activated metabolism pathways related to cysteine, alanine, and glycine (Child et al. 2018). This finding was supported by the upregulation of gene *GOZ73_RS10580*, which encodes ferredoxin.

In addition to amino acid metabolism, nucleotide metabolism also plays a crucial role in the growth of *A. muciniphila* . As an energy‐consuming process, nucleotide metabolism is vital for cell survival and proliferation. The pyrimidine and purine metabolism pathways in *A. muciniphila* remained activated throughout the co‐cultivation with camellia seed oil (Table S8). The upregulated genes, such as *mazG* (encoding nucleoside triphosphate pyrophosphohydrolase), *GOZ73_RS10865* (encoding adenosylcobalamin‐dependent ribonucleoside‐diphosphate reductase), and *GOZ73_RS11610* (encoding adenylosuccinate synthase), played crucial roles in the dynamic balance of nucleotide compounds (Nguyen et al. 2016; Nigo et al. 2023; Schell et al. 2021). Meanwhile, the upregulated d‐erythrose 4‐phosphate suggested the activation of pentose phosphate pathway, which can generate ribose‐5‐phosphate and benefit the nucleotide synthesis (Stincone et al. 2015). The elevated levels of metabolites (e.g., 5′‐adenylic acid, cytidine, deoxyguanosine, and thymine) further confirmed the activation of nucleotide metabolism pathways of *A. muciniphila* under camellia seed oil treatment, thereby facilitating the cell replication of *A. muciniphila* .

The growth of *A. muciniphila* during the exponential growth phase requires multiple rounds of DNA replication and the following transcription and translation (Rolfe et al. 2012). The translation process in ribosome provides cells with various essential proteins for diverse bioactivities. The proliferation of bacteria is strictly limited by ribosome biosynthesis (Donati, Montanaro, and Derenzini 2012). In this study, genes associated with translation, such as *rpsG*, *rpmC*, *rplR*, *proS*, and *gatA* (decoding the ribosome subunits, tRNA ligase, and tRNA amidotransferase), were significantly upregulated under camellia seed oil treatment (Table S9), particularly during the lag growth phase (4 h). This enhancement followed the robust energy metabolism observed in the co‐cultivated *A. muciniphila* , as the bacteria prepared to synthesize essential cellular components.

Additionally, other metabolic pathways, including the biosynthesis of cofactors, the two‐component system, and microbial metabolism in diverse environments, could exhibit promotive effects on the proliferation of *A. muciniphila* co‐cultivated with camellia seed oil. For instance, once *A. muciniphila* was introduced to the environment with camellia seed oil, or the nutrients in the media were about to be depleted, the genes and metabolites associated with microbial metabolism in diverse environment pathway were regulated to adapt to the new environment. In this study, the upregulated gene (e.g., *katE*, encoding catalase HPII) could be responsible for the elevated antioxidant activities. Under oxygen exposure, the accumulation of reactive oxygen species (ROS), such as hydrogen peroxide (H_2_O_2_), can lead to oxidative stress and damage to lipids, proteins, and DNA, potentially resulting in cell death. This is critical for anaerobic bacteria, including *A. muciniphila* , as it greatly influences their viability (Amaretti et al. 2013; Feng and Wang 2020). Catalase is associated with cellular antioxidant defense by decomposing H_2_O_2_, which can modulate the generation of hydroxyl radicals, thus benefiting the proliferation of *A. muciniphila* (Wang et al. 2017). The activated two‐component system in this study (Table 1) could further regulate physiological responses to adapt to the environment (Shaw, Hess, and Weimer 2022). The elevated levels of cofactors, including NADH, l‐ascorbate, and vitamin B2, were essential for cell growth, particularly in maintaining redox homeostasis (Averianova et al. 2020; Senizza et al. 2020). Furthermore, indole derivatives could promote the proliferation of *A. muciniphila* (Yin et al. 2021) and biofilm formation through the quorum sensing signaling pathway (Inaba et al. 2020). The GSEA results (Table 1) and the elevated levels of 3‐indoleacrylic acid and indole‐3‐lactic acid in this study are consistent with these studies.

Overall, the bioactive compounds present in camellia seed oil promoted the energy metabolism in *A. muciniphila* , stimulated the biosynthesis of nucleotides and proteins, thereby facilitating adaptation to the environment and resulting in enhanced proliferation of *A. muciniphila* . However, the underlying molecular mechanisms and the specific bioactive compounds responsible for these effects remain unclear, highlighting the necessity for further comprehensive investigations in future studies.

## 5 Conclusions

This study elucidated that camellia seed oil could enhance the anaerobic proliferation of *A. muciniphila* in vitro. This effect may not be solely due to the presence of fatty acids in camellia seed oil but rather to other bioactive compounds. Transcriptomic and metabolomic profiling indicated that camellia seed oil could modulate the expression of genes and metabolites associated with energy metabolism, nucleotide metabolism, protein synthesis, and environmental adaptation, thereby facilitating the proliferation of *A. muciniphila* . These findings suggest that camellia seed oil exhibits prebiotic‐like effects on *A. muciniphila* . However, the underlying molecular mechanisms and the specific bioactive compounds responsible for these effects remain unclear, highlighting the need for further comprehensive investigations in future studies.

## Author Contributions

**Xi Chen:** conceptualization (lead), data curation (lead), validation (lead), writing – original draft (lead), writing – review and editing (lead). **Yong Zhu:** investigation (lead), methodology (lead), visualization (lead). **Muhammad Mazhar:** data curation (supporting), formal analysis (lead). **Likang Qin:** conceptualization (supporting), funding acquisition (lead), project administration (lead), resources (lead), supervision (lead).

## Ethics Statement

The authors have nothing to report.

## Conflicts of Interest

The authors declare no conflicts of interest.

## Supporting information


Data S1

## Data Availability

The transcriptomic datasets supporting the conclusions of this article are available in the NCBI BioProject repository under the accession number PRJNA1086711.
