# Supplementary material for: Transcriptomic and Metabolomic Insights Into the Prebiotic Potential of Camellia Seed Oil for Enhancing Akkermansia muciniphila Proliferation In Vitro
Source: Food Sci Nutr. 2024 Dec 5;13(1):e4637. doi: 10.1002/fsn3.4637 (PMC11717052; doi:10.1002/fsn3.4637)
Supplement: Supplementary file 1 — Data S1 [file FSN3-13-e4637-s001.docx]

Transcriptomic and Metabolomic Insights into the Prebiotic Potential of Camellia Seed Oil for Enhancing *Akkermansia muciniphila* Proliferation *in vitro*

Xi Chen^1, 2^, Yong Zhu^3^, Muhammad Mazhar^1, 3^, Likang Qin^1, 3*^

1 Key Laboratory of Plant Resource Conservation and Germplasm Innovation in Mountainous Region (Ministry of Education), College of Life Sciences/Institute of Agro-Bioengineering, Guizhou University Guiyang, China

2 Department of Laboratory Medicine, Affiliated Jinyang Hospital, Guizhou Medical University, Guiyang, China

3 School of Liquor and Food Engineering, Guizhou University, Guiyang, China

*CORRESPONDENCE

Likang Qin: lkqin@gzu.edu.cn


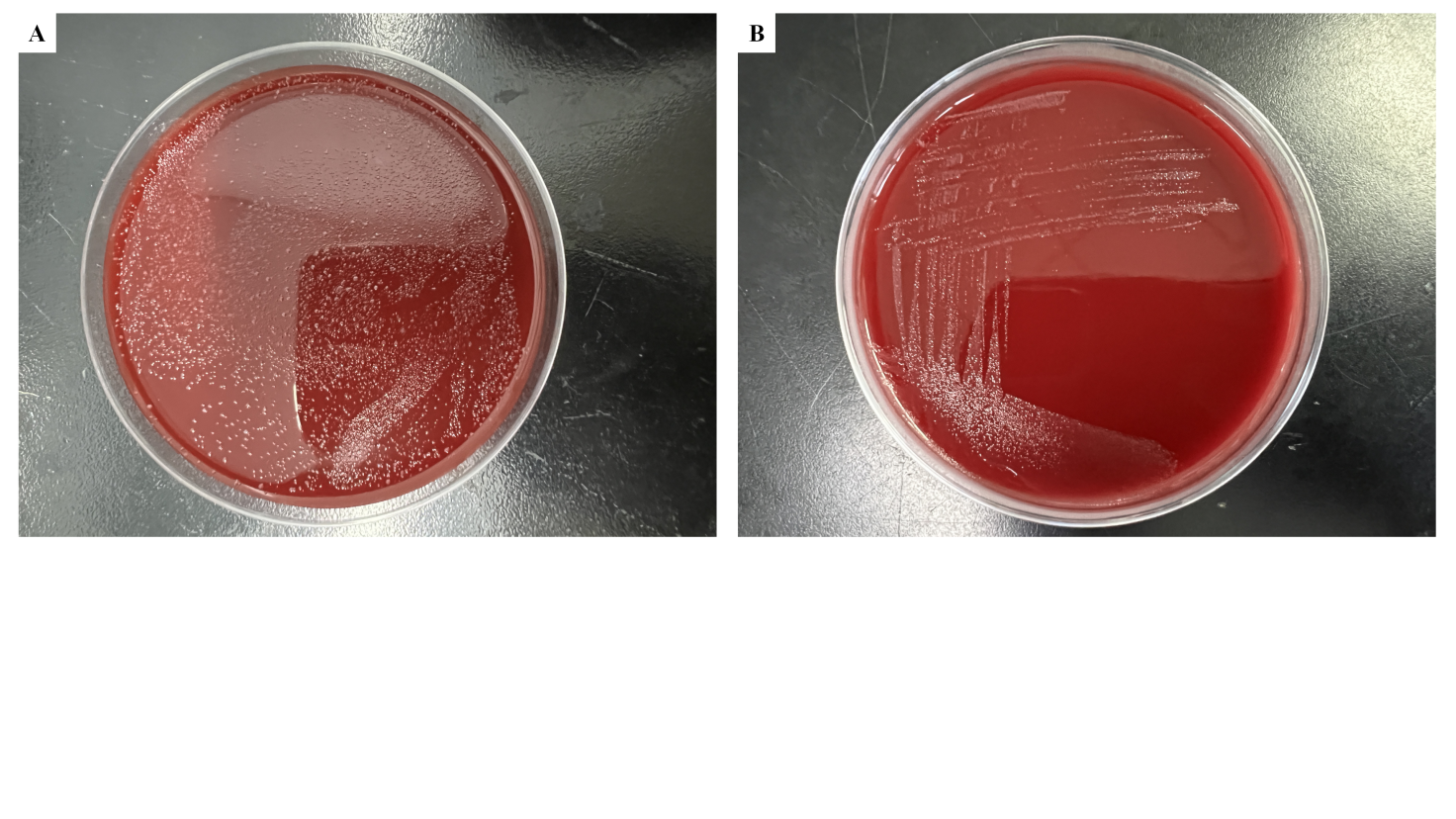


**Fig. S1** *A. muciniphila* was resuscitated on a blood agar plate, exhibiting distinctive growth characterized by colonies with typical morphology of 0.5-1 mm diameter, round shape, opaqueness, smooth texture, and a grayish-white appearance. **(A)** Spread cultivation. **(B)** Streak cultivation.


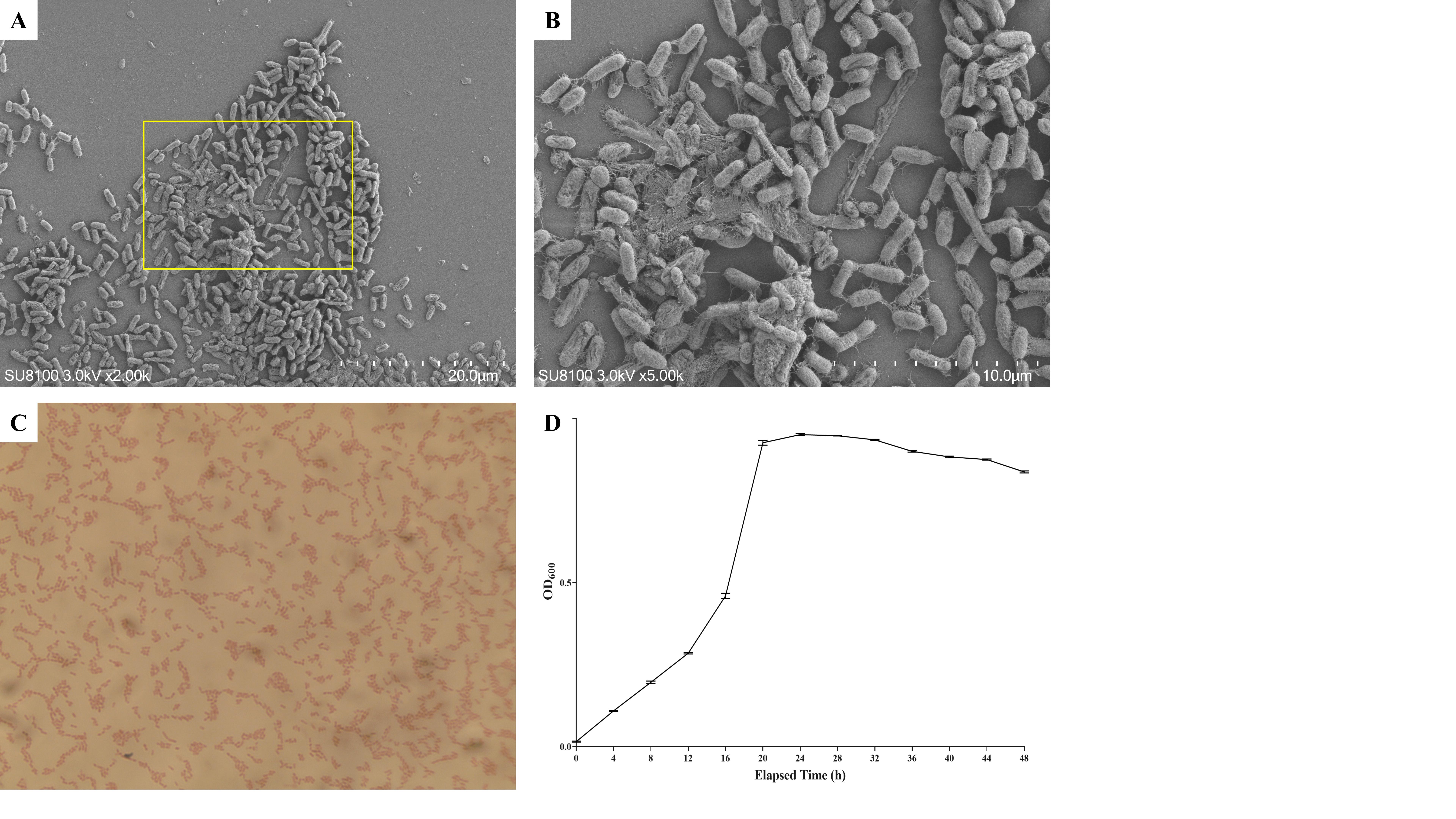


**Fig. S2** Characterization of *A. muciniphila*. The cells exhibited a length of 1 μm by scanning electron microscope observations **(A-B)** and a negative Gram stain appearance **(C)**. The growth curve indicated that *A. muciniphila* reached the stationary growth phase around the 24th hour of cultivation in BHI broth **(D)**, presented as the mean ± SEM (n = 3).


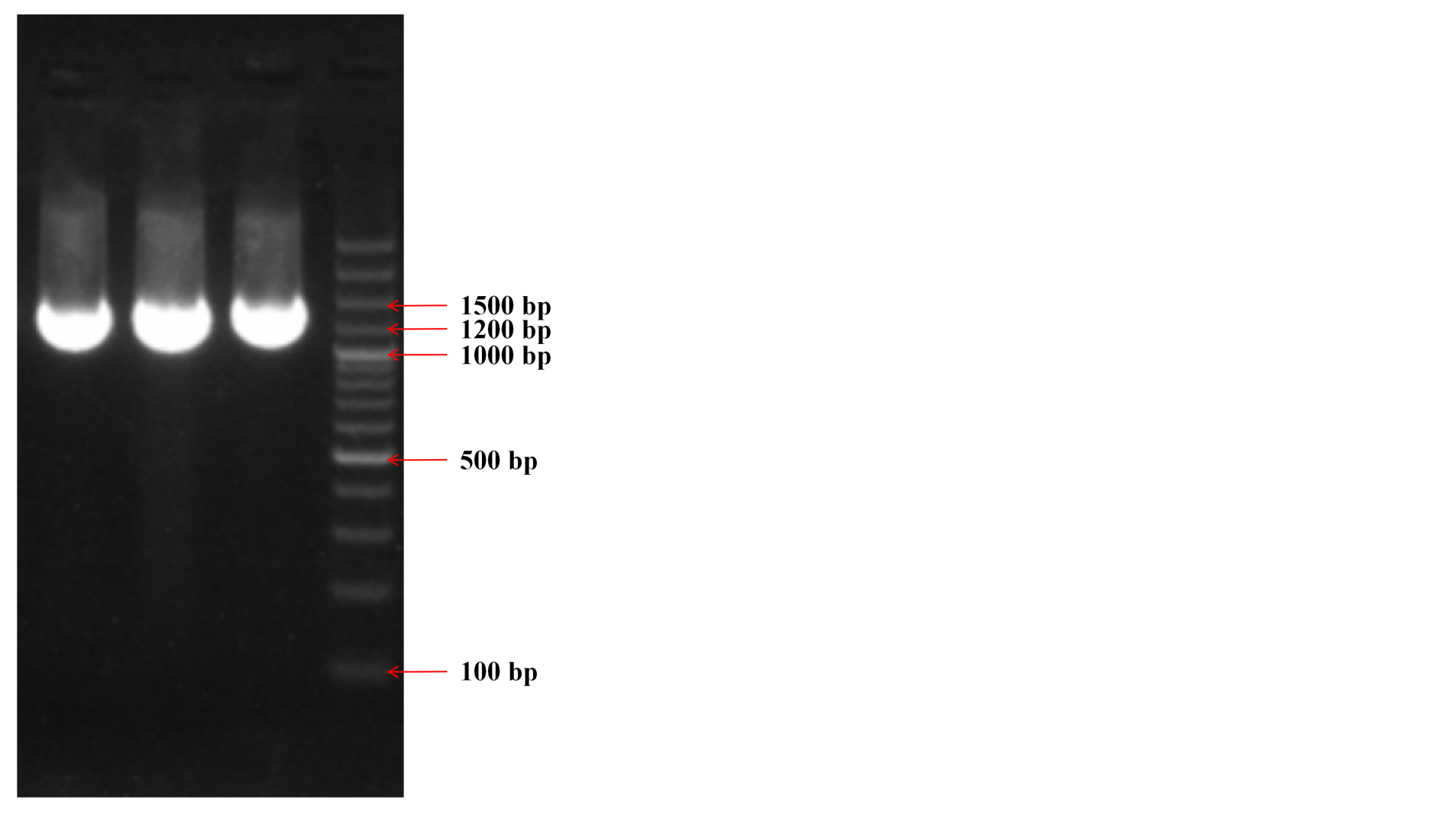


**Fig. S3** 1% agarose gel electrophoresis of genomic DNA extracted from colonies co-cultivated with camellia seed oil (n = 3).


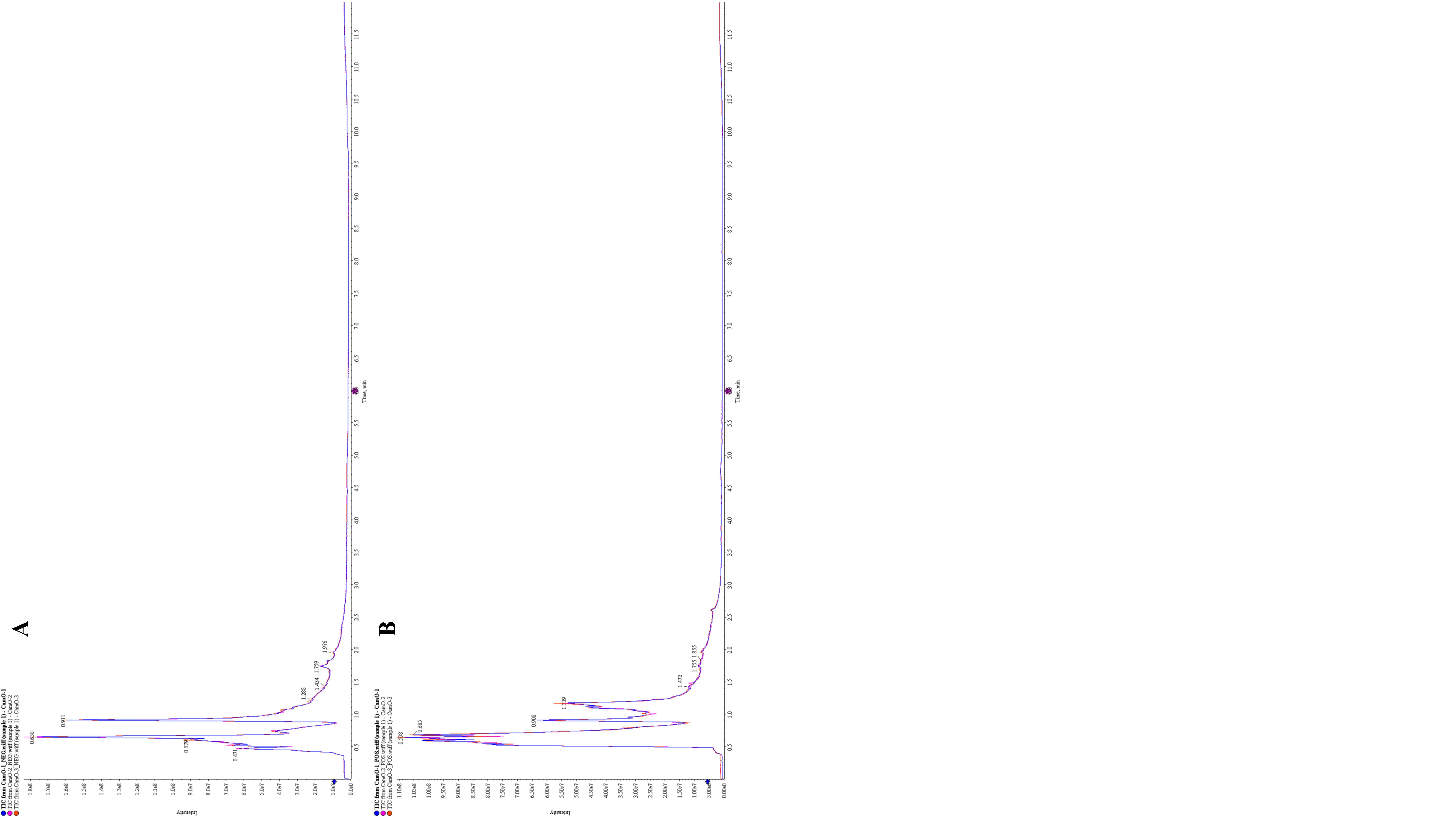


**Fig. S4** Total ion chromatogram of camellia seed oil in negative ion mode **(A)** and positive ion mode **(B)** (n = 3).


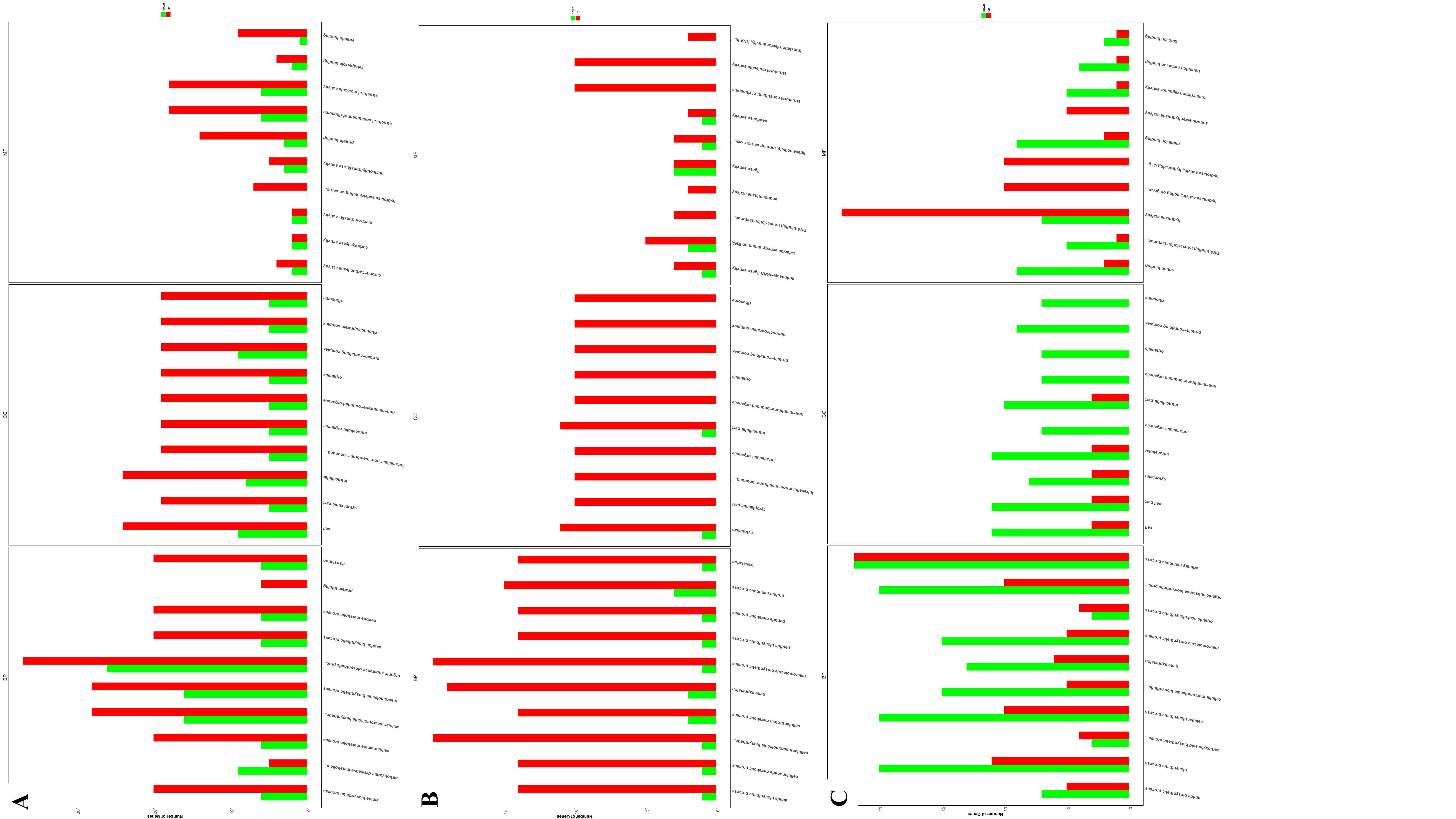


**Fig. S5** GO enrichment diagrams of DEGs of *A. muciniphila* during co-cultivation with camellia seed oil at different time points: **(A)** O4 vs. J4 (4-hour cultivation), **(B)** O16 vs. J16 (16-hour cultivation), and **(C)** O24 vs. J24 (24-hour cultivation) (n = 3). Caption: horizontal axis, GO Term; vertical axis, number of up-regulated (depicted in red) and down-regulated (depicted in green) genes within the respective GO Term.


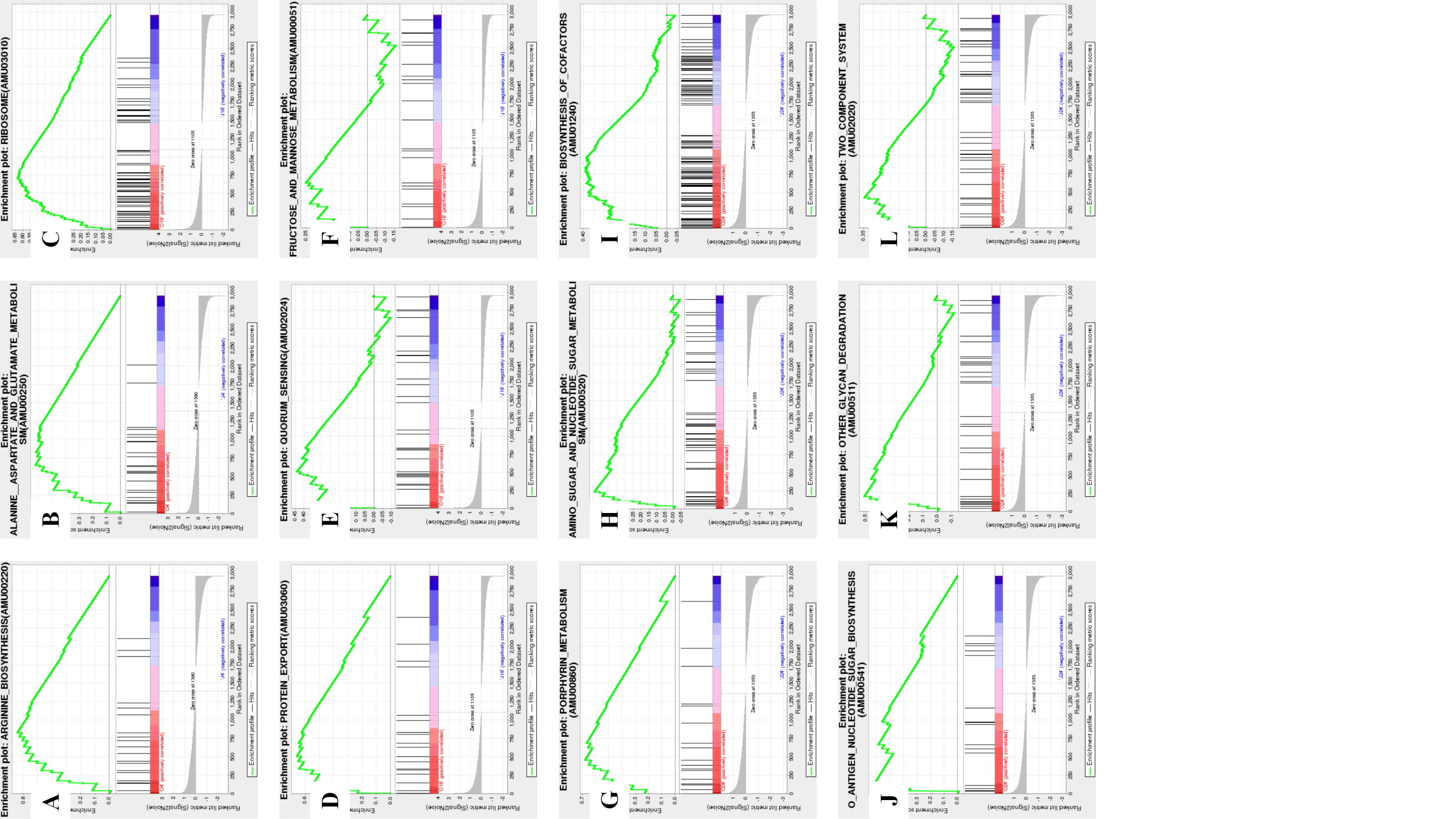


**Fig. S6** GSEA of DEGs of *A. muciniphila* during co-cultivation with camellia seed oil at different time points: **(A-B)** 4-hour co-cultivation; **(C-F)** 16-hour co-cultivation; **(G-L)** 24-hour co-cultivation (n = 3)**.**


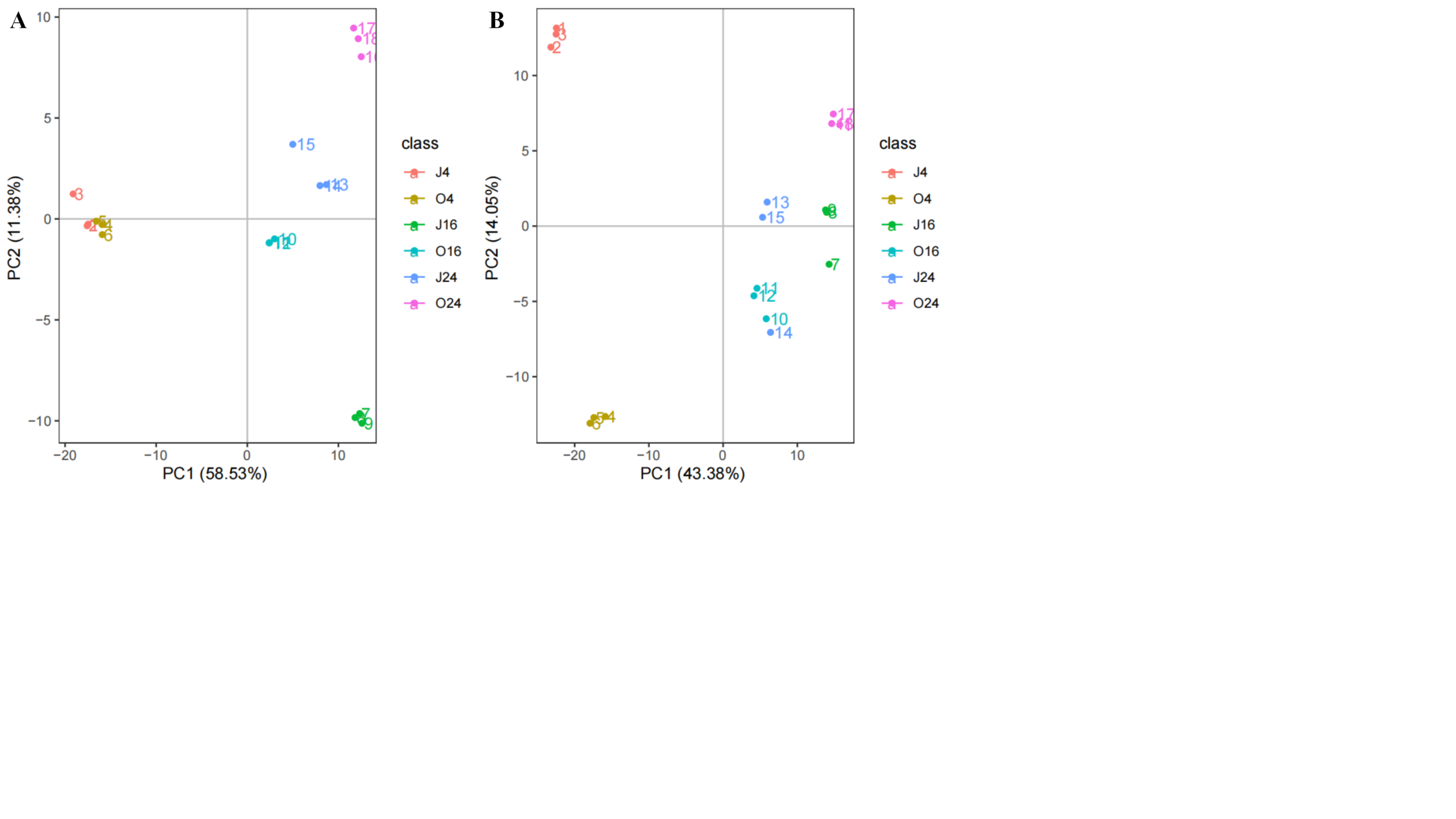


**Fig. S7** PCA of *A. muciniphila* during co-cultivation with camellia seed oil in negative ion mode **(a)** and positive ion mode **(b)** of metabolomic analysis (n = 3).


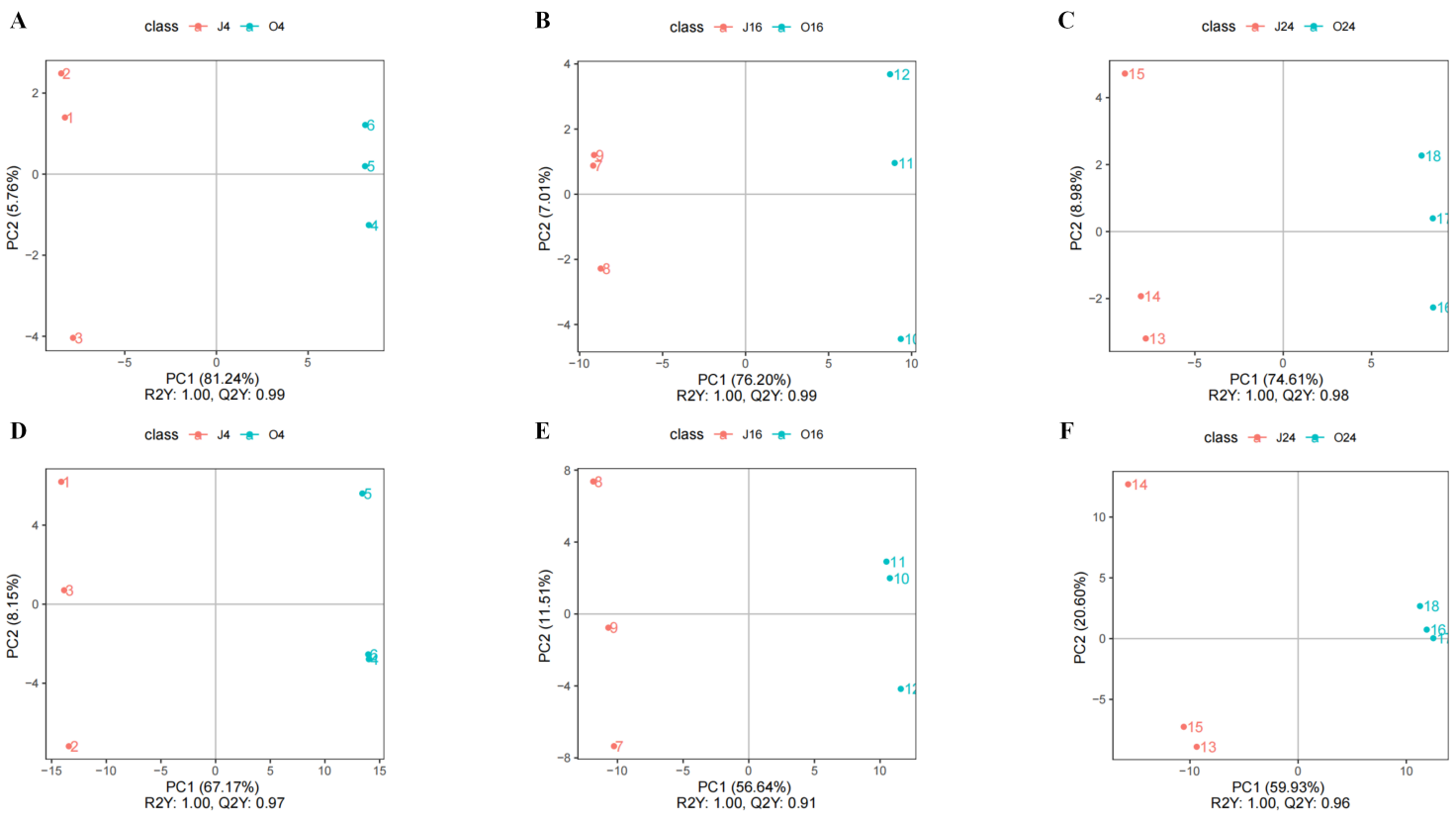


**Fig. S8** PLS-DA scores of *A. muciniphila* during co-cultivation with camellia seed oil at different time points of metabolomic analysis: O4 vs. J4 (4-hour cultivation) in negative **(A)** and positive **(D)** ion mode; O16 vs. J16 (16-hour cultivation) in negative **(B)** and positive **(E)** ion mode; O24 vs. J24 (24-hour cultivation) in negative **(C)** and positive **(F)** ion mode (n = 3).


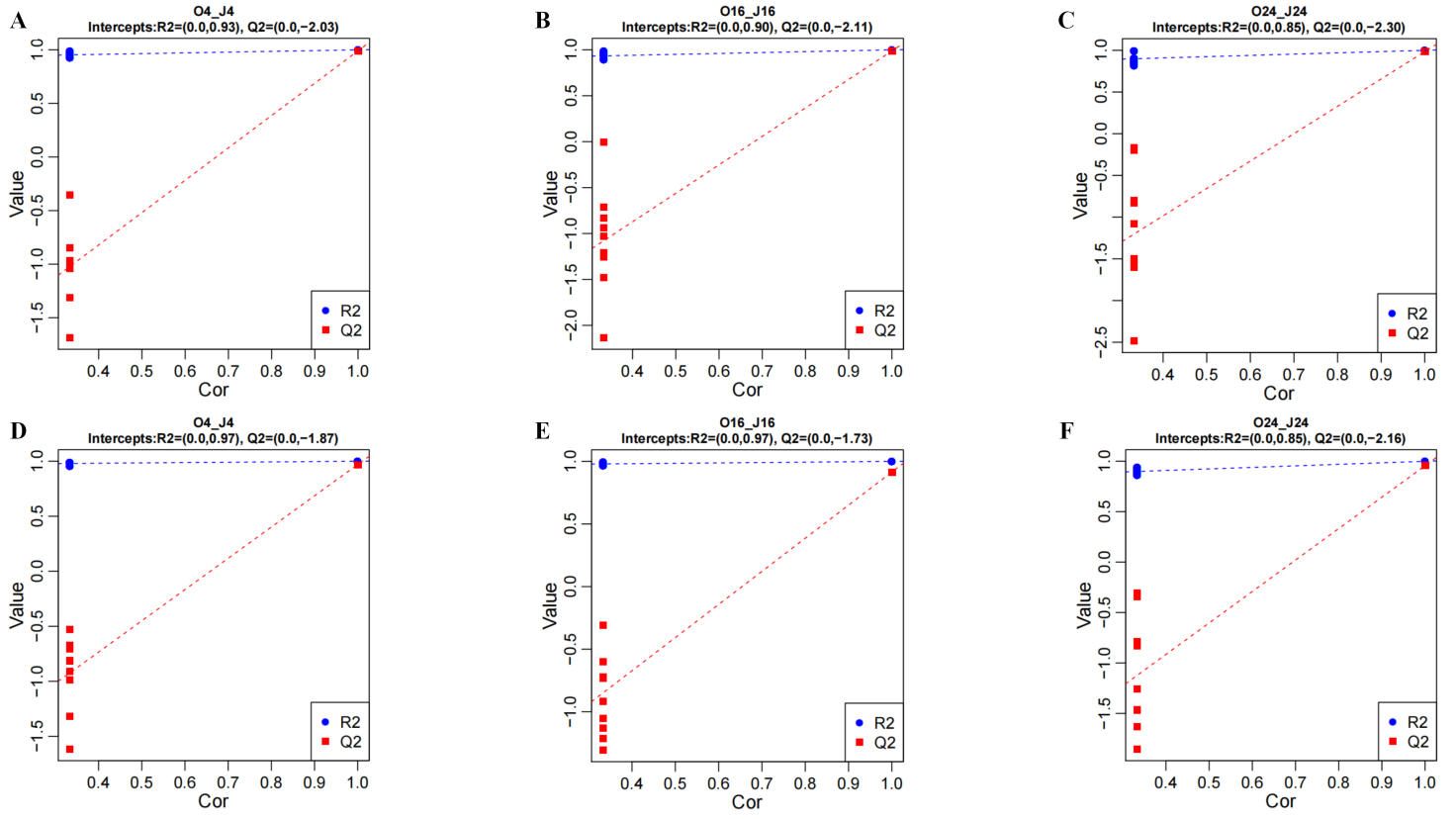


**Fig. S9** Permutation test results for the PLS-DA models of *A. muciniphila* during co-cultivation with camellia seed oil at different time points of metabolomic analysis: O4 vs. J4 (4-hour cultivation) in negative **(A)** and positive **(D)** ion mode, O16 vs. J16 (16-hour cultivation) in negative **(B)** and positive **(E)** ion mode, O24 vs. J24 (24-hour cultivation) in negative **(C)** and positive **(F)** ion mode (n = 3).


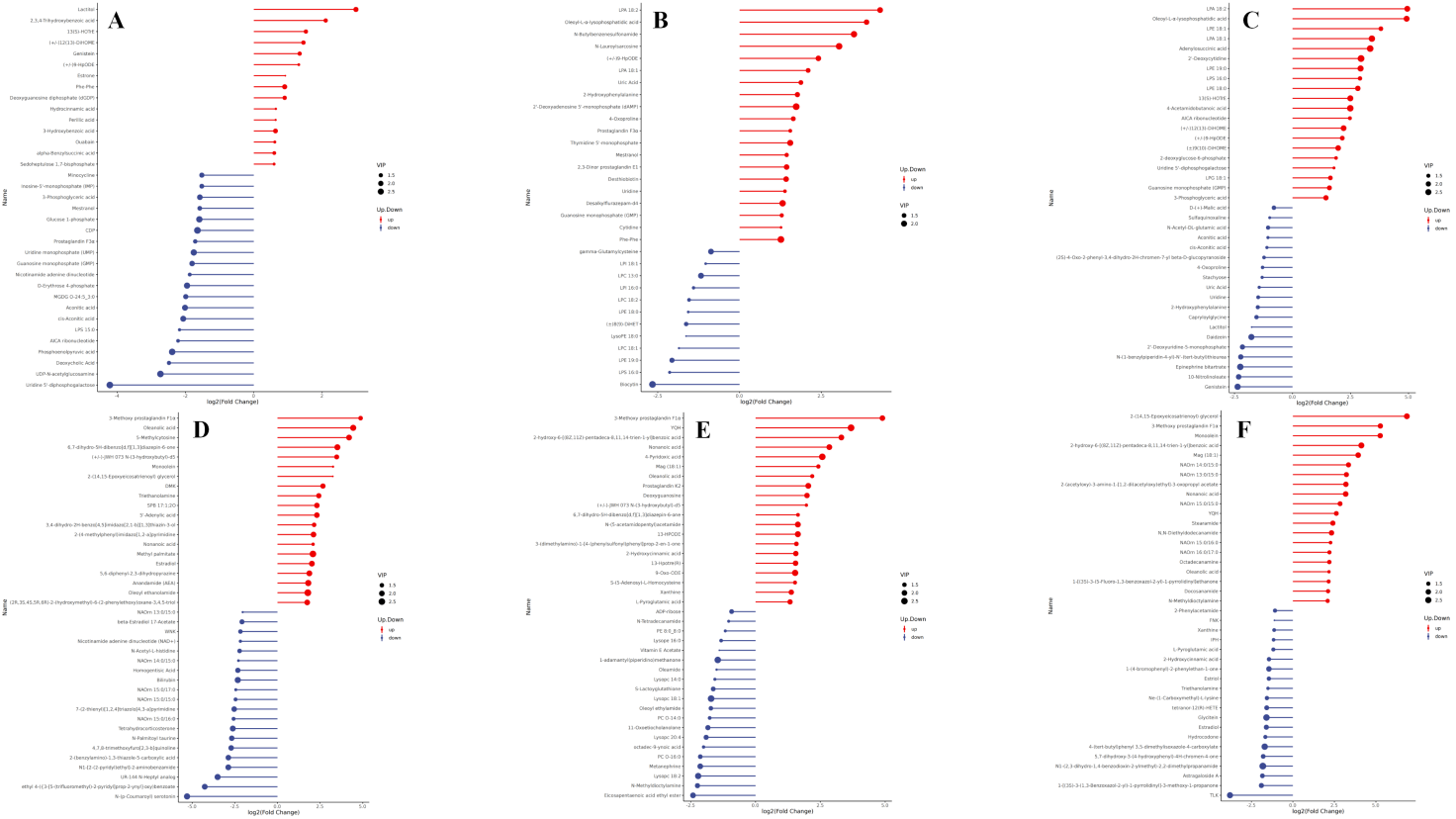


**Fig. S10** Top 20 DEMs of *A. muciniphila* during co-cultivation with camellia seed oil at different time points of metabolomic analysis: O4 vs. J4 (4-hour cultivation) in negative **(A)** and positive **(D)** ion mode, O16 vs. J16 (16-hour cultivation) in negative **(B)** and positive **(E)** ion mode, O24 vs. J24 (24-hour cultivation) in negative **(C)** and positive **(F)** ion mode (n = 3).

**Table S1 Primer sequences for RT-qPCR**

| Gene | Forward | Reverse |
| --- | --- | --- |
| *16S rRNA* | GCTCGTGTCGTGAGATGTTTG | CCACCTTCCTCCCAGTTGAT |
| *GOZ73_RS00180* | GCGGATGTAGCTCAGGGGTAG | GAGCGGATGATGGGATTCG |
| *thiS* | TTGAACATGAAAGGCAAACCC | CGGCGACGATGGAGACAAT |
| *GOZ73_RS07470* | GCGGGTATAGCTTAATGGT | GGAGCGGGTAACGGGAATC |
| *GOZ73_RS10580* | CCTGCATTGACTGCGACCTT | CGGCTGCTTCTTGACATAGGA |
| *GOZ73_RS11860* | AACTGGCATCGGTTCTGGC | AAAAGGAGCGCATTCTGGA |
| *GOZ73_RS09405* | CGGCAGCATTGTTCATCAGA | TGGGAAGGTTCAGCGTGTAA |
| *GOZ73_RS01960* | CCCGTGAAGGTATGCGAGAA | GGAATCCGTATCCGCCACC |
| *GOZ73_RS06700* | GGAAAGAAATGAATAGGCAAGG | CGCAGCACTACGGATGAAC |
| *ykgO* | TCTTTCTTCCTTAGCCTCTATGAA | GCCTTGAACTTGGGATTGC |
| *rpsT* | ACCTCCAAGTTGAAGACCCTCCG | GCTCTTGTAGTTGGCTGCACGATT |

**Table S2** Sequencing results of extracted genomic DNA of colonies co-cultivated with camellia seed oil

| Description | [Max Score](https://blast.ncbi.nlm.nih.gov/Blast.cgi?CMD=Get&ALIGNMENTS=10&ALIGNMENT_VIEW=Pairwise&CONFIG_DESCR=2,3,4,5,6,7,8&DATABASE_SORT=0&DESCRIPTIONS=10&DYNAMIC_FORMAT=on&FIRST_QUERY_NUM=0&FORMAT_OBJECT=Alignment&FORMAT_PAGE_TARGET=&FORMAT_TYPE=HTML&GET_SEQUENCE=yes&I_THRESH=&LINE_LENGTH=60&MASK_CHAR=2&MASK_COLOR=1&NUM_OVERVIEW=10&PAGE=MegaBlast&QUERY_INDEX=0&QUERY_NUMBER=0&RESULTS_PAGE_TARGET=&RID=VFNE5GX0016&SHOW_LINKOUT=yes&SHOW_OVERVIEW=yes&STEP_NUMBER=&ADV_VIEW=off&DISPLAY_SORT=1&HSP_SORT=1) | [Total Score](https://blast.ncbi.nlm.nih.gov/Blast.cgi?CMD=Get&ALIGNMENTS=10&ALIGNMENT_VIEW=Pairwise&CONFIG_DESCR=2,3,4,5,6,7,8&DATABASE_SORT=0&DESCRIPTIONS=10&DYNAMIC_FORMAT=on&FIRST_QUERY_NUM=0&FORMAT_OBJECT=Alignment&FORMAT_PAGE_TARGET=&FORMAT_TYPE=HTML&GET_SEQUENCE=yes&I_THRESH=&LINE_LENGTH=60&MASK_CHAR=2&MASK_COLOR=1&NUM_OVERVIEW=10&PAGE=MegaBlast&QUERY_INDEX=0&QUERY_NUMBER=0&RESULTS_PAGE_TARGET=&RID=VFNE5GX0016&SHOW_LINKOUT=yes&SHOW_OVERVIEW=yes&STEP_NUMBER=&ADV_VIEW=off&DISPLAY_SORT=2&HSP_SORT=1) | [Query Cover](https://blast.ncbi.nlm.nih.gov/Blast.cgi?CMD=Get&ALIGNMENTS=10&ALIGNMENT_VIEW=Pairwise&CONFIG_DESCR=2,3,4,5,6,7,8&DATABASE_SORT=0&DESCRIPTIONS=10&DYNAMIC_FORMAT=on&FIRST_QUERY_NUM=0&FORMAT_OBJECT=Alignment&FORMAT_PAGE_TARGET=&FORMAT_TYPE=HTML&GET_SEQUENCE=yes&I_THRESH=&LINE_LENGTH=60&MASK_CHAR=2&MASK_COLOR=1&NUM_OVERVIEW=10&PAGE=MegaBlast&QUERY_INDEX=0&QUERY_NUMBER=0&RESULTS_PAGE_TARGET=&RID=VFNE5GX0016&SHOW_LINKOUT=yes&SHOW_OVERVIEW=yes&STEP_NUMBER=&ADV_VIEW=off&DISPLAY_SORT=4&HSP_SORT=0) | [E Value](https://blast.ncbi.nlm.nih.gov/Blast.cgi?CMD=Get&ALIGNMENTS=10&ALIGNMENT_VIEW=Pairwise&CONFIG_DESCR=2,3,4,5,6,7,8&DATABASE_SORT=0&DESCRIPTIONS=10&DYNAMIC_FORMAT=on&FIRST_QUERY_NUM=0&FORMAT_OBJECT=Alignment&FORMAT_PAGE_TARGET=&FORMAT_TYPE=HTML&GET_SEQUENCE=yes&I_THRESH=&LINE_LENGTH=60&MASK_CHAR=2&MASK_COLOR=1&NUM_OVERVIEW=10&PAGE=MegaBlast&QUERY_INDEX=0&QUERY_NUMBER=0&RESULTS_PAGE_TARGET=&RID=VFNE5GX0016&SHOW_LINKOUT=yes&SHOW_OVERVIEW=yes&STEP_NUMBER=&ADV_VIEW=off&DISPLAY_SORT=0&HSP_SORT=0) | [Per. Ident](https://blast.ncbi.nlm.nih.gov/Blast.cgi?CMD=Get&ALIGNMENTS=10&ALIGNMENT_VIEW=Pairwise&CONFIG_DESCR=2,3,4,5,6,7,8&DATABASE_SORT=0&DESCRIPTIONS=10&DYNAMIC_FORMAT=on&FIRST_QUERY_NUM=0&FORMAT_OBJECT=Alignment&FORMAT_PAGE_TARGET=&FORMAT_TYPE=HTML&GET_SEQUENCE=yes&I_THRESH=&LINE_LENGTH=60&MASK_CHAR=2&MASK_COLOR=1&NUM_OVERVIEW=10&PAGE=MegaBlast&QUERY_INDEX=0&QUERY_NUMBER=0&RESULTS_PAGE_TARGET=&RID=VFNE5GX0016&SHOW_LINKOUT=yes&SHOW_OVERVIEW=yes&STEP_NUMBER=&ADV_VIEW=off&DISPLAY_SORT=3&HSP_SORT=3) | Accession |
| --- | --- | --- | --- | --- | --- | --- |
| *Akkermansia muciniphila* strain JCM 30893 chromosome, complete genome | 2675 | 1448 | 100 | 0.0 | 100.000 | [CP048438.1](https://www.ncbi.nlm.nih.gov/nucleotide/CP048438.1) |

**Table S3** Nucleotide sequences of extracted genomic DNA of colonies co-cultivated with camellia seed oil

| Species Name | Nucleotide Sequence |
| --- | --- |
| *Akkermansia muciniphila* | GGCTCAGAACGAACGCTGGCGGCGTGGATAAGACATGCAAGTCGAACGAGAGAATTGCTAGCTTGCTAATAATTCTCTAGTGGCGCACGGGTGAGTAACACGTGAGTAACCTGCCCCCGAGAGCGGGATAGCCCTGGGAAACTGGGATTAATACCGCATAGTATCGAAAGATTAAAGCAGCAATGCGCTTGGGGATGGGCTCGCGGCCTATTAGTTAGTTGGTGAGGTAACGGCTCACCAAGGCGATGACGGGTAGCCGGTCTGAGAGGATGTCCGGCCACACTGGAACTGAGACACGGTCCAGACACCTACGGGTGGCAGCAGTCGAGAATCATTCACAATGGGGGAAACCCTGATGGTGCGACGCCGCGTGGGGGAATGAAGGTCTTCGGATTGTAAACCCCTGTCATGTGGGAGCAAATTAAAAAGATAGTACCACAAGAGGAAGAGACGGCTAACTCTGTGCCAGCAGCCGCGGTAATACAGAGGTCTCAAGCGTTGTTCGGAATCACTGGGCGTAAAGCGTGCGTAGGCTGTTTCGTAAGTCGTGTGTGAAAGGCGCGGGCTCAACCCGCGGACGGCACATGATACTGCGAGACTAGAGTAATGGAGGGGGAACCGGAATTCTCGGTGTAGCAGTGAAATGCGTAGATATCGAGAGGAACACTCGTGGCGAAGGCGGGTTCCTGGACATTAACTGACGCTGAGGCACGAAGGCCAGGGGAGCGAAAGGGATTAGATACCCCTGTAGTCCTGGCAGTAAACGGTGCACGCTTGGTGTGCGGGGAATCGACCCCCTGCGTGCCGGAGCTAACGCGTTAAGCGTGCCGCCTGGGGAGTACGGTCGCAAGATTAAAACTCAAAGAAATTGACGGGGACCCGCACAAGCGGTGGAGTATGTGGCTTAATTCGATGCAACGCGAAGAACCTTACCTGGGCTTGACATGTAATGAACAACATGTGAAAGCATGCGACTCTTCGGAGGCGTTACACAGGTGCTGCATGGCCGTCGTCAGCTCGTGTCGTGAGATGTTTGGTTAAGTCCAGCAACGAGCGCAACCCCTGTTGCCAGTTACCAGCACGTGAAGGTGGGGACTCTGGCGAGACTGCCCAGATCAACTGGGAGGAAGGTGGGGACGACGTCAGGTCAGTATGGCCCTTATGCCCAGGGCTGCACACGTACTACAATGCCCAGTACAGAGGGGGCCGAAGCCGCGAGGCGGAGGAAATCCTAAAAACTGGGCCCAGTTCGGACTGTAGGCTGCAACCCGCCTACACGAAGCCGGAATCGCTAGTAATGGCGCATCAGCTACGGCGCCGTGAATACGTTCCCGGGTCTTGTACACACCGCCCGTCACATCATGGAAGCCGGTCGCACCCGAAGTATCTGAAGCCAACCGCAAGGAGGCAGGGTCCTAAGGTGAGACTGGTAACTGGGATGAAGTCGT |

**Table S4** Compounds present in camellia seed oil

| Compound Name | m/z | rt (s) | Average Signal Intensity of Three Duplicate Samples |
| --- | --- | --- | --- |
| delta.8-tetrahydrocannabinol | 193.12 | 59.89 | 16391.72 |
| gamma.-linolenic acid | 279.23 | 57.24 | 1012049.98 |
| (+-)5,6-DHET | 339.25 | 106.67 | 76492.26 |
| (+-)8,9-DHET | 321.24 | 56.55 | 45871.57 |
| (r)-(+)-arachidonyl-1'-hydroxy-2'-propylamide | 362.32 | 73.61 | 252299.31 |
| 1-hexadecanoyl-2-octadecadienoyl-sn-glycero-3-phosphocholine | 758.56 | 150.06 | 5232.95 |
| 1-hexadecylamine | 242.28 | 153.12 | 9477.75 |
| 1-linoleoyl-2-oleoyl-rac-glycerol | 619.52 | 34.25 | 736108.45 |
| 1-monolinoleoyl-rac-glycerol | 355.28 | 36.23 | 136404.72 |
| 1-palmitoyl-2-linoleoyl-rac-glycerol | 615.49 | 32.91 | 366181.89 |
| 1-palmitoyl-2-oleoyl-3-linoleoyl-rac-glycerol | 601.51 | 33.92 | 1104529.82 |
| 1-palmitoyl-2-oleoyl-sn-glycerol | 617.51 | 33.59 | 554078.68 |
| 1-palmitoylglycerol | 313.27 | 34.92 | 171707.42 |
| 1-stearoyl-2-linoleoyl-sn-glycero-3-phospho-l-serine | 810.53 | 76.9 | 31592.8 |
| 1-stearoyl-rac-glycerol | 341.3 | 35.93 | 68137.81 |
| 1,2-dihexadecanoyl-sn-glycerol | 591.49 | 33.59 | 130416.1 |
| 1,2-dilinoleoylglycerol | 599.5 | 33.59 | 348587.76 |
| 1,2-dioleoyl-sn-glycerol | 603.53 | 42.16 | 4376653.82 |
| 1,2-dithiolane-3-pentanamide, n,n'-1,3-propanediylbis- | 473.14 | 35.62 | 10473.57 |
| 12,13-dihydroxy-9z-octadecenoic acid | 315.25 | 82.9 | 77030.59 |
| 13-keto-9z,11e-octadecadienoic acid | 295.22 | 37.53 | 111575.85 |
| 15-deoxy-goyazensolide | 284.33 | 137.39 | 22040.54 |
| 15-ketoprostaglandin a1 | 317.21 | 57.9 | 43406.65 |
| 15-ketoprostaglandin f2.alpha. | 335.22 | 60.51 | 30943.44 |
| 15(s)-15-methylprostaglandin f2.alpha. ethylamide | 378.32 | 168.28 | 13695.9 |
| 16-Hydroxypalmitic acid | 295.22 | 81.53 | 191131.01 |
| 17-octadecynoic acid | 263.23 | 36.89 | 222941.98 |
| 17.alpha.-nandrolone | 549.37 | 57.24 | 30748.91 |
| 18.beta.-glycyrrhetinic acid | 471.34 | 55.89 | 60711.23 |
| 2-linoleoyl-1-palmitoyl-sn-glycero-3-phosphoethanolamine | 575.5 | 34.25 | 313007.22 |
| 2-linoleoylglycerol | 337.27 | 33.59 | 168381.26 |
| 2-methylamino-1-phenylbutane | 133.1 | 33.69 | 14933.12 |
| 2-oleoyl-1-palmitoyl-sn-glycero-3-phosphocholine | 760.58 | 151.11 | 4012.28 |
| 2,4,6-tri-tert-butylaniline | 246.24 | 75.59 | 1827444.19 |
| 21-deoxycortisol | 311.22 | 59.89 | 95017.77 |
| 3-(2-ethylhexoxy)propan-1-amine | 188.2 | 132.93 | 4530.02 |
| 3-acetyl-11-keto-.beta.-boswellic acid | 535.36 | 59.18 | 18751.81 |
| 3-methylbenzyl alcohol | 105.07 | 40.21 | 158588.54 |
| 5-[5-(acetyloxymethyl)-1,2,4a-trimethyl-7-oxo-3,4,8,8a-tetrahydro-2h-naphthalen-1-yl]-3-methylpentanoic acid | 417.21 | 39.54 | 157500.28 |
| 5z,8z,14z-eicosatrienoic acid | 307.26 | 55.2 | 23650.86 |
| 6-[3-[(3,4-dimethoxyphenyl)methyl]-4-methoxy-2-(methoxymethyl)butyl]-4-methoxy-1,3-benzodioxole | 415.21 | 39.54 | 4146507.7 |
| 6-Aminocaproic acid | 114.09 | 46.1 | 3836.33 |
| 6-ketoprostaglandin e1 | 351.21 | 116 | 20666.42 |
| 6-methyl-5-hepten-2-ol | 129.1 | 420.56 | 5979.89 |
| 9-oxo-10e,12z-octadecadienoic acid | 295.22 | 59.18 | 549568.95 |
| all cis-(6,9,12)-Linolenic acid | 339.25 | 56.22 | 86336.74 |
| Allo-protolichesterinic acid | 325.23 | 57.24 | 45515.76 |
| alpha-Linolenic acid | 296.25 | 38.88 | 231670.9 |
| Andrographolide | 333.2 | 59.85 | 18426.12 |
| Angeloylgomisin h | 523.24 | 34.96 | 550.43 |
| Arachidoyl ethanolamide | 356.35 | 60.51 | 511818.07 |
| Asiatic acid | 453.33 | 36.91 | 203788.28 |
| Bassanolide | 926.62 | 68.48 | 44844.27 |
| Beclomethasone | 409.16 | 40.81 | 159643.65 |
| Benzalkonium chloride (c12) | 304.3 | 134.73 | 10066.56 |
| Betaine | 118.08 | 284.29 | 32488.11 |
| Brassicasterol | 297.24 | 82.86 | 120968.79 |
| Canthaxanthin | 565.4 | 56.47 | 34147.17 |
| Cephaeline | 467.31 | 107.72 | 35821.7 |
| Chenodeoxycholate | 393.31 | 35.59 | 82729.19 |
| Chlorhexidine | 490.17 | 34.33 | 10918.17 |
| Cinobufagin | 443.23 | 138.23 | 3777.17 |
| Cis-10-heptadecenoic acid | 233.22 | 57.9 | 46568.44 |
| cis-9-Palmitoleic acid | 277.21 | 80.85 | 84057.62 |
| Cis-vaccenic acid | 265.25 | 36.91 | 238043.92 |
| Cyclovirobuxin d | 372.34 | 83.46 | 9572.63 |
| D,l-n,n-didesmethyl-o-desmethylvenlafaxine | 218.16 | 140.1 | 12687.78 |
| Demissidine | 400.37 | 62.55 | 19643.55 |
| Desogestrel | 293.21 | 60.55 | 229739.35 |
| Dibucaine | 344.22 | 88.92 | 50991.06 |
| Dilinolenin (9c,12c,15c) | 613.48 | 59.54 | 43784.15 |
| Dodeca-2(e),4(e)-dienoic acid | 179.14 | 56.47 | 29815.95 |
| Erucamide | 338.34 | 36.33 | 197762.5 |
| Ethiprole | 398.97 | 287.36 | 14995.62 |
| Gabapentin | 172.13 | 52.54 | 28424.67 |
| Glucose-6-phosphate | 242.98 | 286.52 | 19625.69 |
| Glutathione, oxidized | 613.15 | 502.01 | 11739.22 |
| Glycerol trioleate | 907.77 | 42.15 | 508653 |
| Glycerophosphocholine | 104.11 | 280.01 | 13351.73 |
| Gramine | 130.06 | 68.32 | 10991.55 |
| Hexadecanoic acid | 257.26 | 186.75 | 1670.41 |
| Isoproterenol | 194.12 | 358.59 | 11939.15 |
| Isovaleryl-l-carnitine | 736.5 | 60.51 | 54723.58 |
| Lauramidopropylbetaine | 343.29 | 176.42 | 8747.64 |
| Leupeptin | 427.3 | 57.52 | 14558.55 |
| Linoleic acid | 281.25 | 55.89 | 539437.41 |
| Madecassic acid | 451.32 | 141.97 | 31818.18 |
| Medicagenic acid | 457.32 | 59.89 | 71795.87 |
| Met-Met-Arg | 437.19 | 39.54 | 91311.54 |
| Methyl .gamma.-linolenate | 261.22 | 57.14 | 95379.68 |
| Methyl 2-octynoate | 155.1 | 53.79 | 40380.72 |
| Monoelaidin | 339.29 | 33.59 | 668343.77 |
| Muscone | 239.23 | 35.62 | 2561.26 |
| Myristamine oxide | 258.28 | 151.11 | 447997.1 |
| N-(9-oxodecyl)acetamide | 214.18 | 46.69 | 55614.2 |
| N-dodecylamine | 186.22 | 161.08 | 20003.04 |
| N-lauroyl-d-erythro-sphinganine | 484.47 | 34.89 | 247265.68 |
| N-octadecylamine | 270.31 | 150.74 | 6389.82 |
| N-octanoylsphingosine | 390.36 | 69.75 | 21358.59 |
| N-oleyl-leucine | 396.34 | 73.6 | 12794 |
| N-palmitoyl-d-sphingosine | 256.26 | 69.75 | 159393.29 |
| N-stearoylsphinganine | 568.56 | 35.55 | 685721.8 |
| N,n-dimethylsphingosine | 328.32 | 36.32 | 9374.04 |
| Oleamide | 282.28 | 36.88 | 6640657.5 |
| Oleic acid | 283.26 | 65.15 | 48440.95 |
| Oleoylserotonin | 441.33 | 36.89 | 86313.93 |
| Oxybutynin | 358.22 | 148.54 | 5571.21 |
| Palmitamide | 256.26 | 37.55 | 500242.02 |
| Palmitoylcarnitine | 422.34 | 171.97 | 4967.85 |
| Perifosine | 923.74 | 81.63 | 13173.74 |
| Phenyl salicylate | 215.07 | 51.5 | 1654.06 |
| Picroside i | 475.15 | 35.65 | 1321.79 |
| Pipamperone | 376.26 | 35.55 | 95771.03 |
| Pleiokomenine a | 717.45 | 117.95 | 11617.75 |
| Pro-Trp | 302.31 | 65.2 | 7928355.74 |
| Prostaglandin e1 | 337.23 | 83.54 | 28203.29 |
| Prostaglandin i2 | 353.23 | 127.08 | 13653.37 |
| Pygenic acid c | 451.32 | 36.89 | 110466.03 |
| Saikosaponin d | 437.34 | 36.23 | 124994.15 |
| Salannin | 619.29 | 30.52 | 186.85 |
| Salinomycin | 733.48 | 115.34 | 9103.51 |
| Schizandrin | 455.18 | 34.97 | 2084.33 |
| Sphingosine | 300.29 | 38.27 | 38361.33 |
| Spirostane -2h, + 1o, o-pen-dhex | 709.42 | 127.27 | 18609.15 |
| Stearidonic acid | 277.21 | 57.9 | 410058.59 |
| Stearoylcarnitine | 428.38 | 34.91 | 48927.8 |
| Surfactin b | 1044.68 | 97.82 | 11561.91 |
| Tamoxifen | 372.24 | 143.28 | 6366.27 |
| Tandutinib | 563.35 | 60.53 | 18725.93 |
| Tetradecylamine | 214.25 | 147.89 | 16419.85 |
| Trans-hydroxyperhexiline | 276.28 | 69.75 | 587683.63 |
| Trenbolone | 271.16 | 427.7 | 1051.84 |
| Triacylglycerol 17:0-18:1-18:1 | 890.81 | 50.89 | 93290.27 |
| Triacylglycerol 18:1-18:1-18:2 | 900.79 | 79.52 | 1006044.31 |
| Trimipramine | 295.21 | 53.99 | 469608.72 |
| Tris(hydroxymethyl)aminomethane | 122.08 | 264.32 | 12856.76 |
| Ursolic acid | 439.35 | 36.89 | 330370.61 |
| Valerylfentanyl | 387.23 | 120.4 | 7930.41 |
| Verapamil | 455.31 | 111.64 | 26935.28 |
| Voacamine | 705.41 | 150.45 | 14076.9 |
| Zerumbone | 119.08 | 39.54 | 180160.28 |

m/z, mass to charge ratio; rt, retention time.

**Table S5** Quality assessment of total RNA of *A. muciniphila* co-cultivated with camellia seed oil

| Sample  Name | Concentration (ng/μL) | Volume  (μL) | Total  Quantity (μg) | RIN* |
| --- | --- | --- | --- | --- |
| J4-1 | 60.000 | 32.00 | 1.92000 | 9.40 |
| J4-2 | 47.000 | 32.00 | 1.50400 | 9.60 |
| J4-3 | 22.000 | 32.00 | 0.70400 | 9.50 |
| O4-1 | 42.000 | 32.00 | 1.34400 | 9.30 |
| O4-2 | 18.000 | 32.00 | 0.57600 | 10.00 |
| O4-3 | 9.000 | 57.00 | 0.51300 | 8.60 |
| J16-1 | 140.000 | 32.00 | 4.48000 | 9.30 |
| J16-2 | 43.000 | 32.00 | 1.37600 | 9.40 |
| J16-3 | 38.000 | 32.00 | 1.21600 | 9.50 |
| O16-1 | 188.000 | 32.00 | 6.01600 | 9.20 |
| O16-2 | 81.000 | 32.00 | 2.59200 | 9.30 |
| O16-3 | 206.000 | 32.00 | 6.59200 | 8.00 |
| J24-1 | 40.000 | 32.00 | 1.28000 | 8.60 |
| J24-2 | 34.000 | 32.00 | 1.08800 | 8.90 |
| J24-3 | 41.000 | 32.00 | 1.31200 | 9.00 |
| O24-1 | 45.000 | 32.00 | 1.44000 | 8.80 |
| O24-2 | 21.000 | 32.00 | 0.67200 | 8.70 |
| O24-3 | 121.000 | 32.00 | 3.87200 | 9.00 |

*RIN (RNA Integrity Number) > 7 is considered as qualifying.

**Table S6** Quality assessment of transcriptomic sequencing data

| Sample name | Raw reads | Clean reads | Raw bases | Clean bases | Error rate (%) | Q20 (%) | Q30 (%) | GC content (%) |
| --- | --- | --- | --- | --- | --- | --- | --- | --- |
| J4-1 | 7732466 | 7490208 | 1.2G | 1.1G | 0.03 | 97.92 | 94.25 | 57.10 |
| J4-2 | 7932886 | 7806506 | 1.2G | 1.2G | 0.02 | 98.13 | 94.71 | 56.90 |
| J4-3 | 7957266 | 7812380 | 1.2G | 1.2G | 0.02 | 98.05 | 94.41 | 57.22 |
| O4-1 | 7668730 | 7599494 | 1.2G | 1.1G | 0.03 | 97.78 | 93.73 | 56.84 |
| O4-2 | 6945064 | 6779426 | 1.0G | 1.0G | 0.02 | 98.19 | 94.82 | 57.39 |
| O4-3 | 7662128 | 7515946 | 1.1G | 1.1G | 0.03 | 97.85 | 94.01 | 57.47 |
| J16-1 | 7453388 | 7343124 | 1.1G | 1.1G | 0.03 | 97.89 | 94.12 | 57.05 |
| J16-2 | 7536338 | 7353934 | 1.1G | 1.1G | 0.03 | 97.58 | 93.91 | 56.55 |
| J16-3 | 8898502 | 8787500 | 1.3G | 1.3G | 0.03 | 97.87 | 94.01 | 56.98 |
| O16-1 | 7831854 | 7680360 | 1.2G | 1.2G | 0.02 | 98.15 | 94.74 | 56.68 |
| O16-2 | 7748222 | 7620916 | 1.2G | 1.1G | 0.02 | 98.03 | 94.47 | 56.71 |
| O16-3 | 7655468 | 7386400 | 1.1G | 1.1G | 0.02 | 98.16 | 94.80 | 56.84 |
| J24-1 | 9096682 | 8782204 | 1.4G | 1.3G | 0.03 | 97.83 | 93.97 | 54.75 |
| J24-2 | 7605330 | 7460500 | 1.1G | 1.1G | 0.02 | 97.98 | 94.36 | 54.72 |
| J24-3 | 7649352 | 7468926 | 1.1G | 1.1G | 0.02 | 97.94 | 94.26 | 54.80 |
| O24-1 | 7991030 | 7830068 | 1.2G | 1.2G | 0.02 | 97.94 | 94.35 | 53.95 |
| O24-2 | 7216730 | 7036092 | 1.1G | 1.1G | 0.03 | 97.85 | 94.03 | 54.89 |
| O24-3 | 7326124 | 7100024 | 1.1G | 1.1G | 0.03 | 97.69 | 93.67 | 54.36 |

**Table S7** Key DEGs and DEMs in amino acid metabolism of *A. muciniphila* co-cultivated with camellia seed oil

| Gene (metabolite) Name | Log_2_ Fold Change (time) | Gene Description |
| --- | --- | --- |
| *gcvP* | 0.31 (4 h) | aminomethyl-transferring glycine dehydrogenase |
| *GOZ73_RS06910* | 0.33 (4 h) | glutamine synthetase III |
| *glsA* | 0.41 (4 h) | glutaminase A |
| *argH* | 0.32 (4 h) | argininosuccinate lyase |
| *GOZ73_RS08930* | 0.42 (4 h) | pyridoxal phosphate-dependent aminotransferase |
| *GOZ73_RS09980* | 0.48 (4 h) |  |
| *GOZ73_RS08310* | 0.4 (4 h) | anthranilate synthase component I family protein |
| *GOZ73_RS03210* | 0.36 (4 h) | diaminopimelate dehydrogenase |
| *dapF* | 0.32 (4 h) | diaminopimelate epimerase |
| *argF* | 0.58 (4 h) | ornithine carbamoyltransferase |
| *metK* | 0.44 (4 h) | methionine adenosyltransferase |
| 5-Methylcytosine | 4.167 (4 h) |  |
| L-Tyrosine | 1.77 (16 h) |  |

**Table S8** Key DEGs and DEMs in nucleotide metabolism of *A. muciniphila* co-cultivated with camellia seed oil

| Gene (metabolite) Name | Log_2_ Fold Change (time) | Gene Description |
| --- | --- | --- |
| *GOZ73_RS10865* | 0.48 (4h) | adenosylcobalamin-dependent ribonucleoside-diphosphate reductase |
| *pyrH* | 0.33 (4h) | UMP kinase |
| *GOZ73_RS11610* | 0.37 (4h) | adenylosuccinate synthase |
| *mazG* | 0.45 (4h) | nucleoside triphosphate pyrophosphohydrolase |
| 5'-Adenylic acid | 2.31 (4h)  1.56 (24h) |  |
| deoxyguanosine | 1.98 (16h)  0.94 (24h) |  |
| guanosine monophosphate | 1.27 (16h)  1.36 (24h) |  |
| adenosine | 0.61 (16h) |  |
| guanosine | 0.67 (16h) |  |
| uric acid | 1.88 (16h) |  |
| xanthine | 1.38 (16h) |  |
| uridine | 1.39 (16h) |  |
| cytidine-5'-monophosphate | 0.9 (16h)  0.85 (24h) |  |
| cytidine | 1.27 (16h) |  |
| guanine | 0.86 (24h) |  |
| adenosine 5'-monophosphate | 0.87 (24h) |  |
| adenylosuccinic acid | 3.35 (24h) |  |
| cytosine | 1.39 (24h) |  |
| thymine | 0.69 (24h) |  |
| 2'-deoxycytidine | 2.95 (24h) |  |

**Table S9** Key DEGs and DEMs in translation process of *A. muciniphila* co-cultivated with camellia seed oil

| Gene (metabolite) Name | Log_2_ Fold Change (time) | Gene Description |
| --- | --- | --- |
| *rpsG* | 0.59 (4h) | 30S ribosomal protein S7 |
| *rpsE* | 0.58 (4h) | 30S ribosomal protein S5 |
| *rplO* | 0.52 (4h) | 50S ribosomal protein L15 |
| *rpsC* | 0.53 (4h) | 30S ribosomal protein S3 |
| *rplK* | 0.46 (4h) | 50S ribosomal protein L11 |
| *rplD* | 0.45 (4h) | 50S ribosomal protein L4 |
| *rplU* | 0.43 (4h) | 50S ribosomal protein L21 |
| *rpsB* | 0.36 (4h) | 30S ribosomal protein S2 |
| *rplA* | 0.4 (4h) | 50S ribosomal protein L1 |
| *rpmC* | 0.63 (4h)  0.47 (16h) | 50S ribosomal protein L29 |
| *rplJ* | 0.39 (4h) | 50S ribosomal protein L10 |
| *rplW* | 0.45 (4h) | 50S ribosomal protein L23 |
| *rplB* | 0.39 (4h) | 50S ribosomal protein L2 |
| *rplF* | 0.36 (4h) | 50S ribosomal protein L6 |
| *rpsQ* | 0.44 (4h) | 30S ribosomal protein S17 |
| *rplP* | 0.36 (4h) | 50S ribosomal protein L16 |
| *rplN* | 0.37 (4h) | 50S ribosomal protein L14 |
| *rpsH* | 0.35 (4h) | 30S ribosomal protein S8 |
| *rplC* | 0.33 (4h) | 50S ribosomal protein L3 |
| *rplR* | 0.44 (4h) | 50S ribosomal protein L18 |
| *rpsJ* | 0.42 (4h) | 30S ribosomal protein S10 |
| *rplE* | 0.31 (4h) | 50S ribosomal protein L5 |
| *rplM* | 0.27 (4h) | 50S ribosomal protein L13 |
| *rpsO* | 0.85 (16h) | 30S ribosomal protein S15 |
| *ykgO* | 0.76 (16h) | type B 50S ribosomal protein L36 |
| *rpmG* | 0.48 (16h) | 50S ribosomal protein L33 |
| *GOZ73_RS03900* | 0.34 (16h) | 50S ribosomal protein L24 |
| *rpsT* | 0.46 (16h) | 30S ribosomal protein S20 |
| *rpmH* | 0.52 (16h) | 50S ribosomal protein L34 |
| *proS* | 0.38 (4h) | proline--tRNA ligase |
| *gatA* | 0.38 (4h) | Asp-tRNA(Asn)/Glu-tRNA(Gln) amidotransferase subunit GatA |
| *GOZ73_RS09865* | 0.35 (24h) | glycine--tRNA ligase |
| *argS* | 0.32 (24h) | arginine--tRNA ligase |
| *pheS* | 0.28 (24h) | phenylalanine--tRNA ligase subunit alpha |
